# The prevalence of patient engagement in published trials: a systematic review

**DOI:** 10.1186/s40900-018-0099-x

**Published:** 2018-05-22

**Authors:** Dean Fergusson, Zarah Monfaredi, Kusala Pussegoda, Chantelle Garritty, Anne Lyddiatt, Beverley Shea, Lisa Duffett, Mona Ghannad, Joshua Montroy, M. Hassan Murad, Misty Pratt, Tamara Rader, Risa Shorr, Fatemeh Yazdi

**Affiliations:** 1 Ottawa Hospital Research Institute, Ottawa, ON Canada; 2 Patient Partner SPOR National Steering Committee, Ottawa, ON Canada; 3 Department of Hematology, Ottawa Hospital Research Institute, Ottawa, ON Canada; 4 Amsterdam Public Health Research Institute, Amsterdam, the Netherlands; 5 Evidence-Based Practice Research Program, Mayo Clinic, Rochester, MN USA; 6 Canadian Agency for Drugs and Technologies in Health, Ottawa, ON Canada; 7 The Ottawa Hospital, Ottawa, ON Canada; 8 Centre for Practice-Changing Research, Office L1298a, 501 Smyth Road, Box 201B, Ottawa, ON K1H 8L6 Canada

**Keywords:** Patient engagement, Patient-oriented research, Systematic review, Clinical trials

## Abstract

**Plain English summary:**

With the growing movement to engage patients in research, questions are being asked about who is engaging patients and how they are being engaged. Internationally, research groups are supporting and funding patient-oriented research studies that engage patients in the identification of research priorities and the design, conduct and uptake of research. As we move forward, we need to know what meaningful patient engagement looks like, how it benefits research and clinical practice, and what are the barriers to patient engagement?

We conducted a review of the published literature looking for trials that report engaging patients in the research. We included both randomized controlled trials and non-randomized comparative trials. We looked at these trials for important study characteristics, including how patients were engaged, to better understand the practices used in trials. Importantly, we also discuss the number of trials reporting patient engagement practices relative to all published trials. We found that very few trials report any patient engagement activities even though it is widely supported by many major funding organizations. The findings of our work will advance patient-oriented research by showing how patients can be engaged and by stressing that patient engagement practices need to be better reported.

**Abstract:**

**Background:**

Patient-Oriented Research (POR) is research informed by patients and is centred on what is of importance to them. A fundamental component of POR is that patients are included as an integral part of the research process from conception to dissemination and implementation, and by extension, across the research continuum from basic research to pragmatic trials [J Comp Eff Res 2012, 1:181–94, JAMA 2012, 307:1587–8]. Since POR’s inception, questions have been raised as to how best to achieve this goal.

We conducted a systematic review of randomized controlled trials and non-randomized comparative trials that report engaging patients in their research. Our main goal was to describe the characteristics of published trials engaging patients in research, and to identify the extent of patient engagement activities reported in these trials.

**Methods:**

The MEDLINE®, EMBASE®, Cinahl, PsycINFO, Cochrane Methodology Registry, and Pubmed were searched from May 2011 to June 16th, 2016. Title, abstract and full text screening of all reports were conducted independently by two reviewers. Data were extracted from included trials by one reviewer and verified by a second. All trials that report patient engagement for the purposes of research were included.

**Results:**

Of the 9490 citations retrieved, 2777 were reviewed at full text, of which 23 trials were included. Out of the 23 trials, 17 were randomized control trials, and six were non-randomized comparative trials. The majority of these trials (83%, 19/23) originated in the United States and United Kingdom. The trials engaged a range of 2-24 patients/ community representatives per study. Engagement of children and minorities occurred in 13% (3/23) and 26% (6/23) of trials; respectively. Engagement was identified in the development of the research question, the selection of study outcomes, and the dissemination and implementation of results.

**Conclusions:**

The prevalence of patient engagement in patient-oriented interventional research is very poor with 23 trials reporting activities engaging patients. Research dedicated to determining the best practice for meaningful engagement is still needed, but adequate reporting measures also need to be defined.

**Electronic supplementary material:**

The online version of this article (10.1186/s40900-018-0099-x) contains supplementary material, which is available to authorized users.

## Background

Patient-Oriented Research (POR) is research informed by patients and is centred on what is of importance to them. A fundamental component of POR is that patients are included as an integral part of the research process from conception to dissemination and implementation, and by extension, across the research continuum from basic research to pragmatic trials [1, 2]. This is commonly, but not solely, referred to as patient engagement [3, 4]. While limited evidence exists with regards to the benefits and potential difficulties of integrating patients into the research process [5–8], there is a clear movement towards doing so, especially in Europe and North America [9–13]. As is stated by the Canadian Strategy for Patient-Oriented Research, “patients bring the perspective as ‘experts’ from their unique experience and knowledge gained through living with a condition or illness” [14]. Engaging patients in research therefore increases its quality and, as healthcare providers integrate this research into care, the quality of care will also increase [14].

In addition to the published literature, the concept of patient engagement in research is supported by established and emerging infrastructure at global, national, and institutional levels. Prominent national level organizations include INVOLVE (www.invo.org.uk), which was established in 1996. It is part of, and funded by, the National Institute for Health Research in the UK, to support active public involvement in the National Health Service (NHS), public health and social care research. INVOLVE acts as a national advisory group to bring together expertise, insight and experience in the field of public involvement in research. Their aim is to advance POR as an essential part of the process by which research is identified, prioritized, designed, conducted and disseminated. PCORI (Patient-Centered Outcomes Research Institute) (www.pcori.org) is a non-profit, non-governmental organization located in Washington, DC. Congress authorized the establishment of PCORI in the Patient Protection and Affordable Care Act of 2010. Financially, the organization is working with an estimated budget of $650 million per year (2014–2019). Canada initiated a national Strategy for Patient-Oriented Research (SPOR) (http://www.cihr-irsc.gc.ca/e/41204.html) in 2011.

While methodological guidance does exist on patient-engagement in research [3, 15], the launch of SPOR in Canada has led to questions raised by the research community: what does patient-oriented research look like?; how is research that engages patients being approached?; how should researchers identify and meaningfully engage with patients?; in which elements of the research process can patients contribute?; how do patients and investigators work together?; and what are the barriers and harms to patient engagement for Canadians?

Some of these questions were explored by PCORI in collaboration with the Mayo Clinic in 2011 [8, 16, 17]. Domecq et al. [8] identified that patient engagement in healthcare research occurs largely at the beginning of qualitative research (protocol and agenda setting), but significantly less so in quantitative research.

The objective of our systematic review was to identify randomized controlled trials and non-randomized comparative trials that report engaging patients in their research study. The goal was to determine the number of trials reporting patient engagement activities, and describe their study characteristics, the quantity, and extent of patient engagement activities within each trial. We aimed to identify key characteristics including research context, which patients or community representatives are engaged, and how patients are engaged.

### Patient partnership

This systematic review was conducted in partnership with an experienced patient advisor. She is a patient partner on the Canadian SPOR National Steering Committee. The patient partner was involved in reviewing and amending the protocol to ensure it was in line with SPOR priorities, and interpreting research results through a patient lens. The patient partner was involved in disseminating study results to appropriate patient communities.

## Methods

This systematic review was conducted on the basis of an established protocol. Please see Additional file 1 for the search strategy.

As was stated in the protocol, the definitions of patient, patient engagement, and patient-oriented research are as follows. We defined patient as “individuals with personal experience of a health issue and informal caregivers, including family and friends” [4]. Patient engagement was defined as the “meaningful and active collaboration in governance, priority setting, conducting research and knowledge translation” [4]. Further, we defined patient-oriented research as “a continuum of research that engages patients as partners, focusses on patient-identified priorities and improves patient outcomes”. The research, conducted by multidisciplinary teams in partnership with relevant stakeholders, “aims to apply the knowledge generated to improve healthcare systems and practices” [4].

### Eligibility criteria

#### Inclusion criteria

We have included all clinical trials that reported a patient perspective elicited for the purposes of research. We have included all reports where patients, family members, caregivers and community members provided input, guidance or consultation (or otherwise described contribution) on at least one element of the research process. Elements included topic generation, priority-setting, question refinement, defining outcomes, methods and study design, statistical analysis plan, interpretation of results, and dissemination and implementation of results.

#### Exclusion criteria

We have excluded all systematic reviews, cross-sectional and non-comparative studies, studies that did not provide any details on patient perspectives, studies with no full text available, and those that were non-English or non-French language.

### Search methods

An experienced information specialist developed and conducted a search of MEDLINE®, EMBASE®, Cinahl, PsycINFO, Cochrane Methodology Registry, and PubMed were searched from May 2011 to March 2nd, 2015. This timeframe aligned with the search that was conducted by Domecq et al. [8]. An updated search of MEDLINE EPUB ®, EMBASE®, Cinahl, PsycINFO, Cochrane Methodology Registry, and PubMed was conducted from January 1st, 2015 to June 16th, 2016. Potentially eligible titles and/or abstracts were identified using a combination of subject headings (e.g., “patient centered care”, “patient participation”, and “patient involvement”) and key words (e.g., “consumer”, “stakeholder”, “patient”) (See Additional file 1). Additional trials eligible for inclusion were identified by members of the research team prior to the start of the project and used as ‘seed’ articles when developing the electronic search strategy.

In order to determine the prevalence, the Cochrane Central Register of Controlled Trials (CENTRAL) was referenced. A full description of the creation of CENTRAL, including an explicit explanation of the methods used to conduct the search, is available on the Cochrane Library website [18].

### Screening

**Fig. 1:**
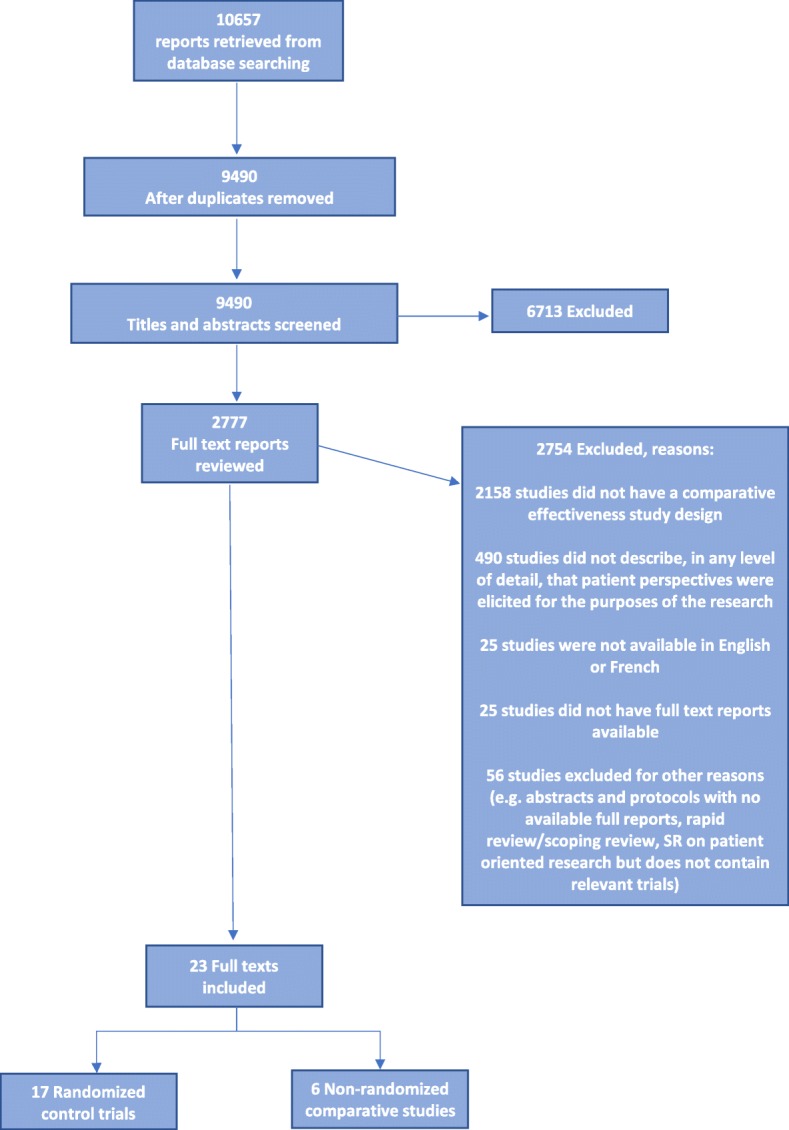
Flow of study reports

Titles and abstracts were screened for potential inclusion using a liberal accelerated approach (i.e., one reviewer to include and two reviewers to exclude [19]) (MP, JM, MG, KP, ZM). Screening of full text reports was completed independently in duplicate by a team of reviewers. All screening disagreements were discussed, with any outstanding disagreements resolved by an independent third reviewer (BS). Data Management software, DistillerSR® [20], was used to manage retrieved records, screen reports, identify and track disagreements. An overview of the results from the screening process is reported using a PRISMA flow diagram (Fig. 1).

### Data extraction

We developed forms for data extraction, according to our a priori protocol and items of interest from the included reports based on Domecq et al. [8]. General characteristics and full data extraction was conducted by one reviewer and verified by a second; a 12% random sample of reports was assessed for accuracy. The following general characteristics were extracted: year of publication; title, aim, study design, country of conduct; topic the study focused on; single or multi-centered trial; ethnicity; number of patient/community representatives engaged in research; total sample size.

The engagement in research was categorized into four components: a) engagement in the development of the research question; b) engagement in selection of outcomes; c) engagement in any other way (e.g. input in design, intervention development, protocol review/approval, recruitment, intervention delivery, input in conduct, input in interpretation); d) dissemination and implementation.

### Analyses

Narrative descriptions were reported for all trials. No formal inferential statistical analyses were conducted. An assessment of the quality and risk of bias of included trials and investigation of meta-bias was not conducted as the purpose of our work focused on identifying the scope and types of patient engagement. Reports were categorized by report type (randomized controlled trial and non-randomized comparative trial).

## Results

### Search and selection results

We retrieved 10,657 reports from electronic searching. These trials were assessed by the study inclusion criteria. Of the 9490 unique title and abstract records retrieved and screened, 2777 full text reports were reviewed for eligibility. A total of 23 reports met the eligibility criteria and were included (Table 1; Additional file 2: Table S2). The trials included 17 randomized-controlled trials and 6 non-randomized trials.

**Table 1:** Study characteristics of included studies

Author, year of publication	Study type	Methods of engagement			
		*Development of research question*	*Selecting outcome*	*Other activities*	*Dissemination and implementation of results*
Mitchell, 2013 [24]	Nonrandomized Comparative Study	Yes	Not reported	Yes	Not reported
Man, M.S., 2015 [25]	Nonrandomized Comparative Study	Not reported	Not reported	Yes	Not reported
Tully, M., 2015 [26]	Nonrandomized Comparative Study	Not reported	Not reported	Yes	Not reported
Goodacre.. 2015 [27]	Nonrandomized Comparative Study	Yes	Not reported	Yes	Yes
Mohatt, G.V., 2014 [28]	Nonrandomized Comparative Study	Not reported	Not reported	Yes	Not reported
Koladooz, F., 2014 [29]	Nonrandomized Comparative Study	Not reported	Not reported	Yes	Not reported
Pearson, 2014 [33]	Randomized Control Trial	Not reported	Not reported	Yes	Not reported
Halanych, 2012 [36]	Randomized Control Trial	Yes	Not reported	Yes	Not reported
Bogart, L.M., 2013 [21]	Randomized Control Trial	Not reported	Not reported	Yes	Yes
Kaholokula, J.K., 2012 [31]	Randomized Control Trial	Not reported	Not Reported	Yes	Yes
Cunningham, S., 2015 [34]	Randomized Control Trial	Not reported	Not reported	Yes	Not reported
Zgibor, J.C., 2016 [38]	Randomized Control Trial	Not reported	Not reported	Yes	Not reported
Bauermeister, J.A., 2015 [30]	Randomized Control Trial	Not reported	Not reported	Yes	Not reported
Huppelschoten, A, G., 2015 [39]	Randomized Control Trial	Not reported	Yes	Yes	Not reported
Ormerod, A.D., 2015 [40]	Randomized Control Trial	Yes	Yes	Yes	Not reported
O’Callaghan, P., 2014 [32]	Randomized Control Trial	Not reported	Not reported	Yes	Not reported
Bowrey, D.J., 2014 [22]	Randomized Control Trial	Not reported	Not reported	Yes	Not reported
Littlewood, E., 2015 [41]	Randomized Control Trial	Not reported	Not reported	Yes	Not reported
McMilan, A., 2015 [42]	Randomized Control Trial	Not reported	Not reported	Yes	Yes
Gaucher, S., 2016 [43]	Randomized Control Trial	Not reported	Not reported	Yes	Not reported
Fraser, R., 2015 [44]	Randomized Control Trial	Not reported	Not reported	Yes	Not reported
Gimeno- Santos, E., 2015 [45]	Randomized Control Trial	Not reported	Not reported	Yes	Not reported
Kattelmann, K., 2014 [23]	Randomized Control Trial	Not reported	Not reported	Yes	Not reported

### Prevalence denominator

The Cochrane Central Register of Controlled Trials (CENTRAL) reported a total of 371,159 published and unpublished randomized and non-randomized controlled trials across several databases, dating back to 2011. Using this figure as a rough estimate, we have effectively found that across all 371,159 clinical trials, only 23 reported patient engagement practices.

### Context

#### Research focus

The majority (82%; 14/17) of randomized controlled trials collected focused on different medical fields. The remaining 3 trials were centred on nutrition and exercise research [21–23]. All of the included non-randomized trials emerged from distinct medical fields including chronic obstructive pulmonary disease (COPD) [24], patient information aids [25], hypertension [26], influenza [27], prevention of suicide and alcohol abuse [28], and nutrition [29].

#### Country of conduct

Included trials originated predominantly in the United States (43%, 10/23). Trials originating in the United Kingdom were also included (39%, 9/23). Other countries (13%, 3/23) included the Netherlands, France, and Spain. Only one study (4%, 1/23) was included that reported patient engagement in research that was conducted in Canada [29].

### Patient engagement setting

#### Size

The included trials reported engaging a cohort rather than a single patient on the research team. The smallest group consisted of 2 patients [25], and the largest consisted of 24 engaged patients/community representatives [24]. Patient representatives are reported as being patients, parents, caregivers, and members of community organizations.

#### Inclusion of ethnic minority, marginalized, and special populations

As reported in Additional file 2, almost half of trials (48%; 11/23) reported the inclusion of ethnic minority and marginalized populations in their study. Six trials (26%, 6/23) reported engaging patients of certain ethnic and racial denominations on their research team in some capacity [26, 28, 30–33].

Engagement with young patients was noted in three trials (13%, 3/23) [21, 28, 30]. In one study, the intervention was geared towards children. Their parents served as proxies and were engaged on the research team [34].

#### Engagement by research stage

**Fig. 2:**
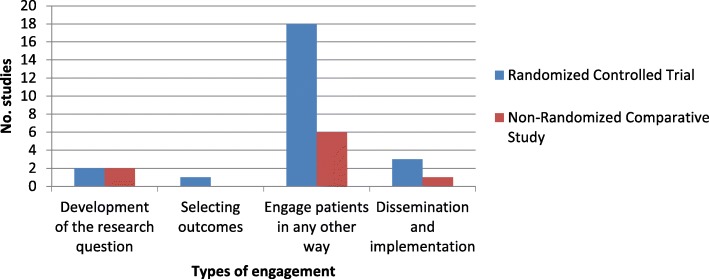
Patient engagement by research design

**Fig. 3:**
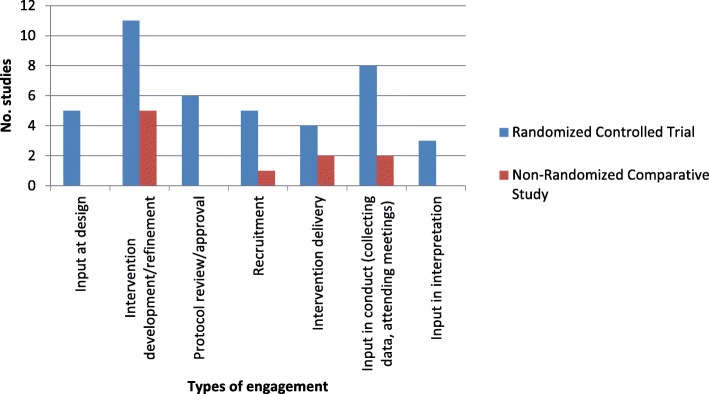
‘Other’ engagement by research design

Figure 2 displays the various types of patient engagement by research design. Our results suggest that research teams were able to engage patients in the development of the research question, the selection of study outcomes, as well as the dissemination and implementation of results. The exception is non-randomized trials where no study engaging patients in selecting outcomes was found. In addition to partnering with patients in developing the research questions, selecting outcomes, or dissemination and implementation of results, all trials reported engagement in a different form. Figure 3 displays “other” forms of patient engagement identified in the included trials. The majority of randomized controlled trials engaged patients in the development or refinement of the study intervention. This is also true of non-randomized comparative trials.

## Discussion

In this review, we identified randomized controlled trials and non-randomized comparative trials that report engagement of patients in research. The results of this review indicated that the prevalence of patient engagement across trials is extremely low in practice. With only 23 trials identified in this report, we estimate that far less than 1% (23/371,159) of clinical trials engage patients meaningfully and actively. The research captured in this review was conducted in various heterogeneous subject areas across the globe. There is not a single phase of research where engagement has not been documented. Generally, patients can contribute to the beginning, middle, and end phases of the research process. In comparison to an earlier systematic review by Domecq et al. [8], it is evident that the answers to basic questions about patient engagement and patient-oriented research have remained largely unchanged or unanswered.

### Difficulties and methodological considerations

In the literature, it is reported that recruitment and retention of patients as partners is a barrier to patient engagement [35]. This is particularly true of research in an acute disease or illness area. In a study on bronchiolitis, authors reported losing a link with patient representatives early in the study [34]. They acknowledge that the short duration of the illness, and frequent misdiagnoses in primary care as potential reasons for this [34]. The team was able to identify patient representatives through admission to the hospital for the remainder of the study [34]. This could be seen as a potential barrier to understanding the perspective of patients in the primary care setting, however.

In several trials, community-based participatory research (CBPR) methods were incorporated into the study design. Benefits of CBPR methods include achieving a more meaningful relationship with the target population and a more effective dissemination and implementation of results [31]. CBPR principles dictate involving community members as integral and equal partners in all research phases [21]. In a trial, authors reported engaging community partners in the development of interventions, intervention testing, data interpretation, and dissemination [21]. Further, in a randomized-controlled trial targeting obesity in the Pacific Islander community, the research team referred to their community partners as “co-researchers”. This team reported community engagement in designing and testing the intervention, delivering the intervention, collecting data, and dissemination and implementation back to the community [31]. Development of the intervention was consistently seen in all trials that reported following a CBPR design [21, 23, 29, 31].

Of the trials where patient engagement activities are reported, our findings suggest that researchers strive to engage patients throughout the length of the research including conduct, implementation, and dissemination [21, 23, 31]. This successful and lasting engagement is potentially due to the advent of the CBPR approach in patient-oriented research. The CBPR approach is a means of working with the community to target their priority concerns. As such, while there exists a great deal of heterogeneity in the fields of research that engage patients, there is an underlying relationship between the trials identified in this review. All focus on prevalent medical conditions that significantly affect key patient-reported priorities.

Domecq and colleagues raised the concern about a lack of comparative effectiveness research in their 2014 review [8]. Their report had not found any comparative effectiveness research on patient engagement methods. The present review has identified that this concern persists and in fact, has led to inconsistent and vague reporting of patient engagement research. Of our identified trials, the quality of reporting on the rationale for patient engagement and the type of engagement chosen is poor. This is potentially due to the absence of legitimate comparison between engagement types to determine best practice. At present, the literature is inconsistent and vague in its reporting.

### Reporting

Patient engagement is reported differently across trials. This irregularity is rooted in both the location of reporting within a research report and in the scope of information provided. Instances of engagement are not clearly distinguished and are often included under various headings in the report. For example, Halanych et al. [36] reported “Community Engagement” under a separate heading in the “Methods” section. This differs from Cunningham et al. [34], who reported “Patient and Public Involvement” under the “Discussion” section. This reporting tends to lack detail and explanation of the rationale behind choosing one method of engagement over another (e.g. focus group vs. community advisory board). A method of gauging the type and degree of engagement (i.e. from passive involvement or tokenism, to active collaboration) in research is therefore also lacking. Particularly in an emerging field, such as patient-oriented research and patient engagement in research, poor reporting leaves knowledge users with an insufficient understanding of how the work was conducted, thus limiting its reproducibility, applicability, and impact. Neglecting to report key details of patient engagement methods and impacts deprives the research community of knowledge that could advance the field. Poor reporting could also be responsible for the low prevalence of patient engagement across clinical trials. Without adequate reporting, we are unable to comment accurately on the number of trials practicing patient engagement and the impact this has on the research.

This concern for reporting is discussed by Staniszewska et al. [37] as well. Staniszewka et al. set out to conduct systematic reviews on the impact of patient and public involvement in research and on healthcare quality. Their findings were incomplete due to the poor quality of reporting methods and variability in reporting found in the literature. In response to this, the team created the Guidance for Reporting Involvement of Patients and Public (GRIPP) checklist [37]. The intention of GRIPP is to standardize and enhance the quality of Patient and Public involvement in research reporting. The team indicated that there is a moral and ethical imperative to report research adequately. GRIPP is the first attempt to identify the most important aspects of good reporting in patient and public involvement work.

### Strengths and limitations

Questions surrounding patient engagement are moving beyond the “why” to the “how”. Our review aimed to understand what patient engagement strategies are being used in trials. The paucity of trials reporting patient engagement highlights the gaps in patient-oriented research conduct and reporting. The narrative synthesis of the findings allows a critical assessment of the literature by identifying common themes in a diverse group of trials.

There are limitations to our study. While we followed systematic review methodology, our team decided not to conduct any quality assessment. PRISMA was not an appropriate framework to use given the type of review we conducted; we aimed to collect all examples of patient engagement reported across trials. The quality of the trial’s conduct and reporting was therefore irrelevant. As Domecq et al. [8] reported as well, conducting quality assessment relates to the outcome of the study, and is inessential to understanding patient engagement strategies used. Further, we are unable to verify the quality or potential for bias of the included trials because patient-oriented research is lacking a tool to assess validity.

A further limitation is in the reporting of patient engagement. Our work is limited by the information reported in the publications. It is possible that research teams did partner with patients, but did not report this in the publication as their focus would be on the results of the trial.

## Conclusions

The number of trials reporting patient engagement remains extremely low despite the presence and promotion of patient-oriented research across the globe. In the trials where patient engagement is reported, we noted patients involved in an array of subject areas, and from the beginning of a study to its completion. In patient-oriented research, ethnic, minority, and marginalized populations are engaged is a means of meaningfully achieving outcomes that are important to these groups. Barriers still exist in patient engagement and comparative data determining the best practices of engagement are needed. We also suggest that a standardized manner of reporting patient engagement (e.g. GRIPP 2) is necessary to facilitate comparisons between patient engagement methods as well as conduct.

## Additional files

Additional file 1: Search Strategy. (PDF 57 kb)

Additional file 2: **Table S2.** Full study characteristics. (PDF 652 kb)

## Abbreviations

CBPR: Community-Based Participatory Research
PCORI: Patient-Centered Outcomes Research Institute
PE: Patient engagement
POR: Patient-Oriented Research
SPOR: Strategy for Patient-Oriented Research
