# Supplementary material for: The prevalence of patient engagement in published trials: a systematic review
Source: Res Involv Engagem. 2018 May 22;4:17. doi: 10.1186/s40900-018-0099-x (PMC5963039; doi:10.1186/s40900-018-0099-x)
Supplement: Supplementary file 1 — Search Strategy. (PDF 57 kb) [file 40900_2018_99_MOESM1_ESM.pdf]

Additional File 1: Search Strategy

Database: Embase Classic+Embase <1947 to 2015 February 26>, Ovid MEDLINE(R) In-Process & Other Non-Indexed Citations and Ovid MEDLINE(R) <1946 to Present>
Search Strategy:
----
1 patient participation/ or consumer participation/ or patient advocacy/ or consumer advocacy/ (133102)
2 patient centered care/ or ((patient* or consumer* or stakeholder* or user* or lay* or client* or citizen* or communit* or public or advoca* or carer* or caregiver* or surrogate* or parent* or relative) adj2 (important or perspective or centered or centred or participa* or collaborat* or partner* or voice* or unvoiced)).mp. (383306)
3 ((patient* or consumer* or stakeholder* or user* or lay* or client* or citizen* or communit* or public or advoca* or carer* or caregiver* or surrogate* or parent* or relative) adj2 (involv* or represent* or consult* or contribut* or engage* or activat* or opinion* or dialog* or partner* or input*)).mp. (320574)
4 *patient participation/ or *consumer participation/ or *patient advocacy/ or *consumer advocacy/ or (patient centered care or ((patient* or consumer* or stakeholder* or user* or lay* or client* or citizen* or communit* or public or advoca* or carer* or caregiver* or surrogate* or parent* or relative) adj2 (important or perspective or centered or centred or participa* or collaborat* or partner* or voice* or unvoiced))).ti,ab. or ((patient* or consumer* or stakeholder* or user* or lay* or client* or citizen* or communit* or public or advoca* or carer* or caregiver* or surrogate* or parent* or relative) adj2 (involv* or represent* or consult* or contribut* or engage* or activat* or opinion* or dialog* or partner* or input*)).ti,ab. or patient-centered care/ (606425)
5 exp evaluation studies as topic/ or exp methods/ (24189109)
6 4 and 5 (379463)
7 6 and (outcome*.mp. or quality of life/ or patient preferences/ or risk assessment/ or patient satisfaction/) (110018)
8 6 and ((utilities or values or empiric* or feedback* or communication*).mp. or health priorities/ or research priorities/) (55420)
9 6 and exp clinical trials as topic/ (12372)
10 6 and (physician-patient relations/ or patient acceptance of health care/) (17364)
11 Health Services Research/ or Needs Assessment/ (97247)
12 6 and 11 (5632)
13 6 and agenda*.mp. (1691)
14 4 and *research design/ and (choice behavior/ or cooperative behavior/) (120)
15 6 and (choice behavior/ or cooperative behavior/) (15089)
16 7 or 8 or 10 or 12 or 13 or 15 (164712)
17 16 and ((panel* or jury or juries or forum).mp. or qualitative research/ or interview*.mp.) (20374)
18 16 and (recruit* or participat* or "focus group*" or instrument* or scale* or questionnaire* or consultant* or questionnaire* or survey* or interview* or "nominal group" or delphi*).mp. (77843)
19 17 or 18 (79318)
20 16 and observation*.mp. (8966)
21 (19 or 20) and exp clinical trial as topic/ (1881)
22 19 or 20 (83343)
23 limit 22 to (consensus development conference or consensus development conference, nih or multicenter study or "research support, american recovery and reinvestment act" or research support, nih, extramural or research support, nih, intramural or research support, non us gov't or research support, us gov't, non phs or research support, us gov't, phs) [Limit not valid in Embase; records were retained] (7554)

24 22 and (*patient satisfaction/ or *consumer satisfaction/ or *patient-center care/ or *patient preferences/) (4299)
25 22 and (technology assessment, biomedical/ or community-based participatory research/ or px.fs.) (3199)
26 21 or 24 or 25 (8616)
27 23 and 2 (5782)
28 26 or 27 (12247)
29 limit 28 to (comment or editorial or interview or introductory journal article or legislation or letter or news or newspaper article or patient education handout or retracted publication or "retraction of publication") [Limit not valid in Embase; records were retained] (122)
30 28 not 29 (12125)
31 limit 30 to humans (11576)
32 31 not animals/ (11506)
33 ((patient* or consumer* or stakeholder* or user* or lay* or client* or citizen* or communit* or public or advoca* or carer* or caregiver* or surrogate* or parent* or relative) adj2 (important or perspective or centered or centred or participa* or collaborat* or partner* or voice* or unvoiced)).ti,ab. (187268)
34 ((patient* or consumer* or stakeholder* or user* or lay* or client* or citizen* or communit* or public or advoca* or carer* or caregiver* or surrogate* or parent* or relative) adj2 (involv* or represent* or consult* or contribut* or engage* or activat* or opinion* or dialog* or partner* or input*)).ti,ab. (255772)
35 (24 or 33 or 34) and 32 (10315)
36 (2012$ or 2013$ or 2014$ or 2015$).ed,dc,em,dd. (8238549)
37 (201105$ or 201106$ or 201107$ or 201108$ or 201109$ or 201110$ or 201111$ or "201112$").dd,dc,ed. (1803999)
38 36 or 37 (9702381)
39 35 and 38 (3907)
40 remove duplicates from 39 (3620)

Database: EBM Reviews - Cochrane Methodology Register <3rd Quarter 2012>
Search Strategy:
----
1 (participat* adj2 research).mp. (163)
2 "CMR: Evaluation methodology - patient involvement".kw. (561)
3 "CMR: Other methodology - patient based outcome measures".kw. (271)
4 (design* or planning or priorit* or agenda* or participat*or decision*).mp. and (2 or 3) (262)
5 (2 or 3) and (perspective* or preference*).mp. (139)
6 5 not 4 (86)
7 limit 6 to yr="2011 -Current" (5)

Database: PsycINFO <1806 to February Week 4 2015>
Search Strategy:
----
1 client participation/ (1455)
2 experimentation/ or exp consumer research/ or exp interdisciplinary research/ or exp qualitative research/ or exp experimental design/ or exp methodology/ (180275)
3 1 and 2 (173)
4 1 and (priorit* or participatory or engage* or planning or design* or perspective* or preference*).mp. (835)
5 3 or 4 (914)

6 limit 5 to (all journals and human) (785)
7 1 and (agenda* or involve*).mp. (533)
8 limit 7 to (all journals and human) (448)
9 6 or 8 (922)
10 9 and outcome*.mp. (229)
11 9 and (2 or methodol*.mp.) (178)
12 10 or 11 (362)
13 *client participation/ and 12 (296)
14 limit 13 to yr="2011 - 2015" (103)

Cinahl

Friday, February 27, 2015 2:23:40 PM

#	Query	Limiters/Expanders	Results
S11	S8 and S9	Limiters - Exclude MEDLINE records<br>Search modes - Boolean/Phrase	309
S10	S8 and S9	Search modes - Boolean/Phrase	1,522
S9	method*	Search modes - Boolean/Phrase	681,868
S8	S2 AND (S6 OR S7)	Search modes - Boolean/Phrase	3,350
S7	(MH "Study Design+")	Search modes - Boolean/Phrase	546,559
S6	(MH "Research+") OR (MH "Behavioral Research") OR (MH "Medical Practice, Research-Based")	Search modes - Boolean/Phrase	1,058,438
S5	S2 and S4	Search modes - Boolean/Phrase	130
S4	(MH "Action Research")	Search modes - Boolean/Phrase	3,553
S3	(MH "Patient Centered Care")	Search modes - Boolean/Phrase	13,603
S2	(MM "consumer participation" OR "consumer advocacy")	Search modes - Boolean/Phrase	8,403
S1	input* OR perspective* OR involve* OR planning OR design* OR outcome*	Search modes - Boolean/Phrase	692,044

Pubmed Search – March 02, 2015

("clinical trials as topic"[MeSH Terms] OR "practice guidelines as topic"[MeSH Terms]) OR "health services research"[MeSH Terms] AND ("patient participation/methods"[MAJR] OR "consumer participation/methods"[MAJR]) Filters: Publication date from 2011/00/01
