# Supplementary material for: The prevalence of patient engagement in published trials: a systematic review
Source: Res Involv Engagem. 2018 May 22;4:17. doi: 10.1186/s40900-018-0099-x (PMC5963039; doi:10.1186/s40900-018-0099-x)
Supplement: Supplementary file 2 — Table S2. Full study characteristics. (PDF 652 kb) [file 40900_2018_99_MOESM2_ESM.pdf]

ADDITIONAL FILE 2

Table 2. Full study characteristics of included studies.

† Denotes where excerpts are taken verbatim from study.

Study	Country of conduct	Title	Multi-centre/ single centre	Sample Size	Field/ Topic	Ethnic/ Minority populations	Number of patients/ community representatives engaged in research	Methods of Engagement†			
								Development of research question	*Selecting outcome*	*Other activities*	*Dissemination and Implementation of results*
Pearson, 2014 [33]	USA	Capacity building from the inside out: Development and evaluation of a CITI ethics certification training module for American Indian and Alaska native community researchers	Multi-center	44	Human subject training module	American Indian and Alaska Native	4	Not reported	Not reported	During our first expert panel review with the AI/AN community members, we reviewed all three modules and community members discussed preference and importance of each module.	Not reported

ADDITIONAL FILE 2

Table 2. Full study characteristics of included studies.

† Denotes where excerpts are taken verbatim from study.

**Study**	**Country of conduct**	**Title**	**Multi-centre/ single centre**	**Sample Size**	**Field/ Topic**	**Ethnic/ Minority populations**	**Number of patients/ community representatives engaged in research**	**Methods of Engagement†**			
								Development of research question	*Selecting outcome*	*Other activities*	*Dissemination and Implementation of results*
										Members prioritized the assessing risk and benefit module as they believed this was where researchers often made the biggest mistakes. Also they stated this was where community researchers	

ADDITIONAL FILE 2

Table 2. Full study characteristics of included studies.

† Denotes where excerpts are taken verbatim from study.

**Study**	**Country of conduct**	**Title**	**Multi-centre/ single centre**	**Sample Size**	**Field/ Topic**	**Ethnic/ Minority populations**	**Number of patients/ community representatives engaged in research**	**Methods of Engagement†**			
								Development of research question	*Selecting outcome*	*Other activities*	*Dissemination and Implementation of results*
										are often referred to in reviewing research protocols. The Community Panel provided the first level of review. They made recommendations for changes that would meet the needs of local AI/AN	

ADDITIONAL FILE 2

Table 2. Full study characteristics of included studies.

† Denotes where excerpts are taken verbatim from study.

**Study**	**Country of conduct**	**Title**	**Multi-centre/ single centre**	**Sample Size**	**Field/ Topic**	**Ethnic/ Minority populations**	**Number of patients/ community representatives engaged in research**	**Methods of Engagement†**			
								Development of research question	*Selecting outcome*	*Other activities*	*Dissemination and Implementation of results*
										communities, bring AI/AN culture and lived experience into the revision process and most importantly, identified AI/AN individual and community level risk and benefits. The	

ADDITIONAL FILE 2

Table 2. Full study characteristics of included studies.

† Denotes where excerpts are taken verbatim from study.

**Study**	**Country of conduct**	**Title**	**Multi-centre/ single centre**	**Sample Size**	**Field/ Topic**	**Ethnic/ Minority populations**	**Number of patients/ community representatives engaged in research**	**Methods of Engagement†**			
								Development of research question	*Selecting outcome*	*Other activities*	*Dissemination and Implementation of results*
										investigators and the panel reviewed the core element matrix and then reviewed the module section by section. Changes to the original module were made. In the final, fifth step we sent the final version	

ADDITIONAL FILE 2

Table 2. Full study characteristics of included studies.

† Denotes where excerpts are taken verbatim from study.

**Study**	**Country of conduct**	**Title**	**Multi-centre/ single centre**	**Sample Size**	**Field/ Topic**	**Ethnic/ Minority populations**	**Number of patients/ community representatives engaged in research**	**Methods of Engagement†**			
								Development of research question	*Selecting outcome*	*Other activities*	*Dissemination and Implementation of results*
										out to all panel members for their review. Only minor editorial comments were suggested.	
Mitchell, 2013 [24]	UK	The development and pilot testing of	Multi-center	37	chronic obstructive	Not reported	24	The pulmonary rehabilit	Not reported	The pulmonary rehabilitation team,	Not reported

ADDITIONAL FILE 2

Table 2. Full study characteristics of included studies.

† Denotes where excerpts are taken verbatim from study.

**Study**	**Country of conduct**	**Title**	**Multi-centre/ single centre**	**Sample Size**	**Field/ Topic**	**Ethnic/ Minority populations**	**Number of patients/ community representatives engaged in research**	**Methods of Engagement†**			
								Development of research question	*Selecting outcome*	*Other activities*	*Dissemination and Implementation of results*
		the Self-management Programme of Activity, Coping and Education for Chronic Obstructive Pulmonary Disease (SPACE for COPD)			pulmonary disease			ation team, patients, and health care professionals with expertise in particular topics were involved in the initial development of the		patients, and health care professionals with expertise in particular topics were involved in the initial development of the content and this was further refined through collaboration with	

ADDITIONAL FILE 2

Table 2. Full study characteristics of included studies.

† Denotes where excerpts are taken verbatim from study.

**Study**	**Country of conduct**	**Title**	**Multi-centre/ single centre**	**Sample Size**	**Field/ Topic**	**Ethnic/ Minority populations**	**Number of patients/ community representatives engaged in research**	**Methods of Engagement†**			
								Development of research question	*Selecting outcome*	*Other activities*	*Dissemination and Implementation of results*
								content and this was further refined through collaboration with professionals and patients. Once a draft manual was developed, FGs were		professionals and patients. Once a draft manual was developed, FGs were conducted to review the manual content and determine preferences for the delivery of such a program based	

ADDITIONAL FILE 2

Table 2. Full study characteristics of included studies.

† Denotes where excerpts are taken verbatim from study.

**Study**	**Country of conduct**	**Title**	**Multi-centre/ single centre**	**Sample Size**	**Field/ Topic**	**Ethnic/ Minority populations**	**Number of patients/ community representatives engaged in research**	**Methods of Engagement†**			
								Development of research question	*Selecting outcome*	*Other activities*	*Dissemination and Implementation of results*
								conducted to review the manual content and determine preferences for the delivery of such a program based around a manual to		around a manual to support self-management. The FG schedule was structured to ensure new topics could arise; minor revisions were made to it after the first two FGs.	

ADDITIONAL FILE 2

Table 2. Full study characteristics of included studies.

† Denotes where excerpts are taken verbatim from study.

Study	Country of conduct	Title	Multi-centre/ single centre	Sample Size	Field/ Topic	Ethnic/ Minority populations	Number of patients/ community representatives engaged in research	Methods of Engagement†			
								Development of research question	*Selecting outcome*	*Other activities*	*Dissemination and Implementation of results*
								support self-management. The FG schedule was structured to ensure new topics could arise; minor revisions were made to it after the first			

ADDITIONAL FILE 2

Table 2. Full study characteristics of included studies.

† Denotes where excerpts are taken verbatim from study.

Study	Country of conduct	Title	Multi-centre/ single centre	Sample Size	Field/ Topic	Ethnic/ Minority populations	Number of patients/ community representatives engaged in research	Methods of Engagement†			
								Development of research question	*Selecting outcome*	*Other activities*	*Dissemination and Implementation of results*
								two FGs.			
Halanych, 2012 [36]	USA	Recruitment of a rural, southern, predominantly African-American population into a diabetes self-management trial	Multiple sites	384	diabetes self-management	African-American	Not reported	Prior to developing and submitting the study proposal for funding, study investigators carried out community	Not reported	Study investigators solicited input on study design as well as advice on recruitment approaches	Not reported

ADDITIONAL FILE 2

Table 2. Full study characteristics of included studies.

† Denotes where excerpts are taken verbatim from study.

**Study**	**Country of conduct**	**Title**	**Multi-centre/ single centre**	**Sample Size**	**Field/ Topic**	**Ethnic/ Minority populations**	**Number of patients/ community representatives engaged in research**	**Methods of Engagement†**			
								Development of research question	*Selecting outcome*	*Other activities*	*Dissemination and Implementation of results*
								engagement activities by partnering with an ongoing community coalition established for cancer prevention improvement.			

ADDITIONAL FILE 2

Table 2. Full study characteristics of included studies.

† Denotes where excerpts are taken verbatim from study.

**Study**	**Country of conduct**	**Title**	**Multi-centre/ single centre**	**Sample Size**	**Field/ Topic**	**Ethnic/ Minority populations**	**Number of patients/ community representatives engaged in research**	**Methods of Engagement†**			
								Development of research question	*Selecting outcome*	*Other activities*	*Dissemination and Implementation of results*
								Coalition meetings had consistently included calls for diabetes programs, prompting this proposal, and when the study team received			

ADDITIONAL FILE 2

Table 2. Full study characteristics of included studies.

† Denotes where excerpts are taken verbatim from study.

**Study**	**Country of conduct**	**Title**	**Multi-centre/ single centre**	**Sample Size**	**Field/ Topic**	**Ethnic/ Minority populations**	**Number of patients/ community representatives engaged in research**	**Methods of Engagement†**			
								Development of research question	*Selecting outcome*	*Other activities*	*Dissemination and Implementation of results*
								notice of funding, they collaborated with this coalition to hold a discussion group with community members. With this discussion			

ADDITIONAL FILE 2

Table 2. Full study characteristics of included studies.

† Denotes where excerpts are taken verbatim from study.

Study	Country of conduct	Title	Multi-centre/ single centre	Sample Size	Field/ Topic	Ethnic/ Minority populations	Number of patients/ community representatives engaged in research	Methods of Engagement†			
								Development of research question	*Selecting outcome*	*Other activities*	*Dissemination and Implementation of results*
								group, study investigators<br><br>solicited input on study design as well as advice on recruitment<br><br>approaches			

ADDITIONAL FILE 2

Table 2. Full study characteristics of included studies.

† Denotes where excerpts are taken verbatim from study.

**Study**	**Country of conduct**	**Title**	**Multi-centre/ single centre**	**Sample Size**	**Field/ Topic**	**Ethnic/ Minority populations**	**Number of patients/ community representatives engaged in research**	**Methods of Engagement†**			
								Development of research question	*Selecting outcome*	*Other activities*	*Dissemination and Implementation of results*
Bogart, L.M., 2013 [21]	USA	A Randomized Controlled Trial of Students for Nutrition and eXercise: A Community-Based Participatory Research Study	Multiple sites	2997	Nutrition and Exercise	Not reported	Not reported	Not reported	Not reported	Consistent with CBPR principles [18], school district administrators served on the study leadership team and were integral and equal partners in all research phases, including intervention	Consistent with CBPR principles [18], school district administrators served on the study leadership team and were integral and equal partners in all research phases,

ADDITIONAL FILE 2

Table 2. Full study characteristics of included studies.

† Denotes where excerpts are taken verbatim from study.

**Study**	**Country of conduct**	**Title**	**Multi-centre/ single centre**	**Sample Size**	**Field/ Topic**	**Ethnic/ Minority populations**	**Number of patients/ community representatives engaged in research**	**Methods of Engagement†**			
								Development of research question	*Selecting outcome*	*Other activities*	*Dissemination and Implementation of results*
										development, intervention testing, data interpretation, and dissemination. A community advisory board (CAB) composed of local community and academic experts in	including intervention development, intervention testing, data interpretation, and dissemination. CBPR methods throughout all stages of the research, which

ADDITIONAL FILE 2

Table 2. Full study characteristics of included studies.

† Denotes where excerpts are taken verbatim from study.

**Study**	**Country of conduct**	**Title**	**Multi-centre/ single centre**	**Sample Size**	**Field/ Topic**	**Ethnic/ Minority populations**	**Number of patients/ community representatives engaged in research**	**Methods of Engagement†**			
								Development of research question	*Selecting outcome*	*Other activities*	*Dissemination and Implementation of results*
										adolescent obesity prevention (e.g., from LAUSD administration, parent groups, Los Angeles County's health department, youth-serving organizations, and research institutions) provided	contributed to community partner investment and led to ongoing efforts toward wide-scale program dissemination. For example, the use of CBPR during the

ADDITIONAL FILE 2

Table 2. Full study characteristics of included studies.

† Denotes where excerpts are taken verbatim from study.

**Study**	**Country of conduct**	**Title**	**Multi-centre/ single centre**	**Sample Size**	**Field/ Topic**	**Ethnic/ Minority populations**	**Number of patients/ community representatives engaged in research**	**Methods of Engagement†**			
								Development of research question	*Selecting outcome*	*Other activities*	*Dissemination and Implementation of results*
										critical input and direction in regular in-person meetings.	intervention development and pilot testing phases contributed to the long-term sustainability of some of the environmental aspects of SNaX, through

ADDITIONAL FILE 2

Table 2. Full study characteristics of included studies.

† Denotes where excerpts are taken verbatim from study.

**Study**	**Country of conduct**	**Title**	**Multi-centre/ single centre**	**Sample Size**	**Field/ Topic**	**Ethnic/ Minority populations**	**Number of patients/ community representatives engaged in research**	**Methods of Engagement†**			
								Development of research question	*Selecting outcome*	*Other activities*	*Dissemination and Implementation of results*
											policy changes both within the school district and the state. Specifically, following the pilot's success at increasing cafeteria servings of sliced/bite-sized

ADDITIONAL FILE 2

Table 2. Full study characteristics of included studies.

† Denotes where excerpts are taken verbatim from study.

**Study**	**Country of conduct**	**Title**	**Multi-centre/ single centre**	**Sample Size**	**Field/ Topic**	**Ethnic/ Minority populations**	**Number of patients/ community representatives engaged in research**	**Methods of Engagement†**			
								Development of research question	*Selecting outcome*	*Other activities*	*Dissemination and Implementation of results*
											fruits. Furthermore, a Community board advisory member shared with a state-elected official our pilot study finding that key school leaders said that free

ADDITIONAL FILE 2

Table 2. Full study characteristics of included studies.

† Denotes where excerpts are taken verbatim from study.

**Study**	**Country of conduct**	**Title**	**Multi-centre/ single centre**	**Sample Size**	**Field/ Topic**	**Ethnic/ Minority populations**	**Number of patients/ community representatives engaged in research**	**Methods of Engagement†**			
								Development of research question	*Selecting outcome*	*Other activities*	*Dissemination and Implementation of results*
											drinking water could not be made available with the school meal and helped to draft legislation, informed by our formative work. . In addition, the school district is presently

ADDITIONAL FILE 2

Table 2. Full study characteristics of included studies.

† Denotes where excerpts are taken verbatim from study.

**Study**	**Country of conduct**	**Title**	**Multi-centre/ single centre**	**Sample Size**	**Field/ Topic**	**Ethnic/ Minority populations**	**Number of patients/ community representatives engaged in research**	**Methods of Engagement†**			
								Development of research question	*Selecting outcome*	*Other activities*	*Dissemination and Implementation of results*
											committed to wide-scale, ongoing dissemination of the program and is working with the research team to develop a sustainable model to implement SNaX across

ADDITIONAL FILE 2

Table 2. Full study characteristics of included studies.

† Denotes where excerpts are taken verbatim from study.

**Study**	**Country of conduct**	**Title**	**Multi-centre/ single centre**	**Sample Size**	**Field/ Topic**	**Ethnic/ Minority populations**	**Number of patients/ community representatives engaged in research**	**Methods of Engagement†**			
								Development of research question	*Selecting outcome*	*Other activities*	*Dissemination and Implementation of results*
											the district.
Kaholokula, J.K., 2012 [31]	USA	A Family and Community Focused Lifestyle Program Prevents Weight Regain in Pacific Islanders: A Pilot Randomized	Multiple sites	144	Obesity	Pacific Islanders, such as Native Hawaiians, Samoans, Chuukese, and Filipinos	Not reported	Not reported	Not Reported	Guiding the design of our pilot RCT was a CBPR approach in which the POP's community partners worked side-by-side with the academic partners in designing	"As shared earlier, the community partners identified the research topic and provided input into how the CBPR partnership would

ADDITIONAL FILE 2

Table 2. Full study characteristics of included studies.

† Denotes where excerpts are taken verbatim from study.

**Study**	**Country of conduct**	**Title**	**Multi-centre/ single centre**	**Sample Size**	**Field/ Topic**	**Ethnic/ Minority populations**	**Number of patients/ community representatives engaged in research**	**Methods of Engagement†**			
								Development of research question	*Selecting outcome*	*Other activities*	*Dissemination and Implementation of results*
		Controlled Trial								the PLP O and in determining the study design as described in detail by Nacapoy et al. (2008) and by Mau et al. (2010). "We employed a CBPR approach whereby community members served as	be structured and how the study would be designed and implemented. They also own their own data and determine how the information is disseminated in their respective

ADDITIONAL FILE 2

Table 2. Full study characteristics of included studies.

† Denotes where excerpts are taken verbatim from study.

**Study**	**Country of conduct**	**Title**	**Multi-centre/ single centre**	**Sample Size**	**Field/ Topic**	**Ethnic/ Minority populations**	**Number of patients/ community representatives engaged in research**	**Methods of Engagement†**			
								Development of research question	*Selecting outcome*	*Other activities*	*Dissemination and Implementation of results*
										co-researchers (with co-equal decision making) in all aspects of designing and testing the intervention; in delivering the interventions via community-peer educators	communities. "

ADDITIONAL FILE 2

Table 2. Full study characteristics of included studies.

† Denotes where excerpts are taken verbatim from study.

**Study**	**Country of conduct**	**Title**	**Multi-centre/ single centre**	**Sample Size**	**Field/ Topic**	**Ethnic/ Minority populations**	**Number of patients/ community representatives engaged in research**	**Methods of Engagement†**			
								Development of research question	*Selecting outcome*	*Other activities*	*Dissemination and Implementation of results*
										within their respective communities; and in having community researchers collect baseline and outcome data based on standardized protocol"<br><br>From Nacapoey "Through a	

ADDITIONAL FILE 2

Table 2. Full study characteristics of included studies.

† Denotes where excerpts are taken verbatim from study.

**Study**	**Country of conduct**	**Title**	**Multi-centre/ single centre**	**Sample Size**	**Field/ Topic**	**Ethnic/ Minority populations**	**Number of patients/ community representatives engaged in research**	**Methods of Engagement†**			
								Development of research question	*Selecting outcome*	*Other activities*	*Dissemination and Implementation of results*
										series of meetings facilitated by the Community and Academic Co-Directors, the community-academic partnership negotiated a mutually acceptable research design and protocol that	

ADDITIONAL FILE 2

Table 2. Full study characteristics of included studies.

† Denotes where excerpts are taken verbatim from study.

**Study**	**Country of conduct**	**Title**	**Multi-centre/ single centre**	**Sample Size**	**Field/ Topic**	**Ethnic/ Minority populations**	**Number of patients/ community representatives engaged in research**	**Methods of Engagement†**			
								Development of research question	*Selecting outcome*	*Other activities*	*Dissemination and Implementation of results*
										engaged the community partners as well as maintained scientific rigor. The PILI `Ohana Project partners struggled with the scientific perspective and experimental design issues, such as	

ADDITIONAL FILE 2

Table 2. Full study characteristics of included studies.

† Denotes where excerpts are taken verbatim from study.

Study	Country of conduct	Title	Multi-centre/ single centre	Sample Size	Field/ Topic	Ethnic/ Minority populations	Number of patients/ community representatives engaged in research	Methods of Engagement†			
								Development of research question	*Selecting outcome*	*Other activities*	*Dissemination and Implementation of results*
										using a randomized controlled trial (RCT) protocol, in which participants are randomly assigned to either the intervention being evaluated or to a control group. From the community perspective,	

ADDITIONAL FILE 2

Table 2. Full study characteristics of included studies.

† Denotes where excerpts are taken verbatim from study.

**Study**	**Country of conduct**	**Title**	**Multi-centre/ single centre**	**Sample Size**	**Field/ Topic**	**Ethnic/ Minority populations**	**Number of patients/ community representatives engaged in research**	**Methods of Engagement†**			
								Development of research question	*Selecting outcome*	*Other activities*	*Dissemination and Implementation of results*
										intervention needed to ensure that no participant was denied or delayed in receiving a possibly effective intervention. In balancing these 2 perspectives, the partners agreed upon a RCT design that	

ADDITIONAL FILE 2

Table 2. Full study characteristics of included studies.

† Denotes where excerpts are taken verbatim from study.

**Study**	**Country of conduct**	**Title**	**Multi-centre/ single centre**	**Sample Size**	**Field/ Topic**	**Ethnic/ Minority populations**	**Number of patients/ community representatives engaged in research**	**Methods of Engagement†**			
								Development of research question	*Selecting outcome*	*Other activities*	*Dissemination and Implementation of results*
										ensured all participants would receive an active intervention while still being able to scientifically test the efficacy of the weight loss maintenance intervention ....To build the communitie	

ADDITIONAL FILE 2

Table 2. Full study characteristics of included studies.

† Denotes where excerpts are taken verbatim from study.

**Study**	**Country of conduct**	**Title**	**Multi-centre/ single centre**	**Sample Size**	**Field/ Topic**	**Ethnic/ Minority populations**	**Number of patients/ community representatives engaged in research**	**Methods of Engagement†**			
								Development of research question	*Selecting outcome*	*Other activities*	*Dissemination and Implementation of results*
										s' capacity to engage in research and deliver community-based interventions, it was collectively decided that all the interventions would be delivered, and all data maintained, by community-peer educators	

ADDITIONAL FILE 2

Table 2. Full study characteristics of included studies.

† Denotes where excerpts are taken verbatim from study.

Study	Country of conduct	Title	Multi-centre/ single centre	Sample Size	Field/ Topic	Ethnic/ Minority populations	Number of patients/ community representatives engaged in research	Methods of Engagement†			
								Development of research question	*Selecting outcome*	*Other activities*	*Dissemination and Implementation of results*
										and staff from each of the 5 community sites"<br><br>"In this CBPR partnership, the community partners were completely and equally (e.g., individual budget management) involved	

ADDITIONAL FILE 2

Table 2. Full study characteristics of included studies.

† Denotes where excerpts are taken verbatim from study.

Study	Country of conduct	Title	Multi-centre/ single centre	Sample Size	Field/ Topic	Ethnic/ Minority populations	Number of patients/ community representatives engaged in research	Methods of Engagement†			
								Development of research question	*Selecting outcome*	*Other activities*	*Dissemination and Implementation of results*
										in all aspects of the research project, which a paucity of projects is able to do in practice. As shared earlier, the community partners identified the research topic and provided input into	

ADDITIONAL FILE 2

Table 2. Full study characteristics of included studies.

† Denotes where excerpts are taken verbatim from study.

**Study**	**Country of conduct**	**Title**	**Multi-centre/ single centre**	**Sample Size**	**Field/ Topic**	**Ethnic/ Minority populations**	**Number of patients/ community representatives engaged in research**	**Methods of Engagement†**			
								Development of research question	*Selecting outcome*	*Other activities*	*Dissemination and Implementation of results*
										how the CBPR partnership would be structured and how the study would be designed and implemented. They also own their own data and determine how the information is disseminate	

ADDITIONAL FILE 2

Table 2. Full study characteristics of included studies.

† Denotes where excerpts are taken verbatim from study.

Study	Country of conduct	Title	Multi-centre/ single centre	Sample Size	Field/ Topic	Ethnic/ Minority populations	Number of patients/ community representatives engaged in research	Methods of Engagement†			
								Development of research question	*Selecting outcome*	*Other activities*	*Dissemination and Implementation of results*
										d in their respective communities. The active involvement of communities for which the research findings are to benefit is vital to developing effective and sustainable community engaged	

ADDITIONAL FILE 2

Table 2. Full study characteristics of included studies.

† Denotes where excerpts are taken verbatim from study.

Study	Country of conduct	Title	Multi-centre/ single centre	Sample Size	Field/ Topic	Ethnic/ Minority populations	Number of patients/ community representatives engaged in research	Methods of Engagement†			
								Development of research question	*Selecting outcome*	*Other activities*	*Dissemination and Implementation of results*
										interventions to eliminate health disparities."	
Cunningham, S., 2015 [34]	UK	Bronchiolitis of Infancy Discharge Study (BIDS): a multicentre, parallel-group, double-blind, randomised controlled,	Multiple sites	615	Bronchiolitis	Not reported	Not reported	Not reported	Not reported	In the study set-up stages we engaged with a parent representative to guide protocol development. Unfortunately, that link was lost early in the	Not reported

ADDITIONAL FILE 2

Table 2. Full study characteristics of included studies.

† Denotes where excerpts are taken verbatim from study.

Study	Country of conduct	Title	Multi-centre/ single centre	Sample Size	Field/ Topic	Ethnic/ Minority populations	Number of patients/ community representatives engaged in research	Methods of Engagement†			
								Development of research question	*Selecting outcome*	*Other activities*	*Dissemination and Implementation of results*
		equivalence trial with economic evaluation								study and we were unable to engage appropriate patient and public involvement, possibly as acute bronchiolitis is a relatively short-duration illness for many and is often not given a labelled	

ADDITIONAL FILE 2

Table 2. Full study characteristics of included studies.

† Denotes where excerpts are taken verbatim from study.

**Study**	**Country of conduct**	**Title**	**Multi-centre/ single centre**	**Sample Size**	**Field/ Topic**	**Ethnic/ Minority populations**	**Number of patients/ community representatives engaged in research**	**Methods of Engagement†**			
								Development of research question	*Selecting outcome*	*Other activities*	*Dissemination and Implementation of results*
										diagnosis in primary care. In our prestudy feasibility assessment, we were fortunate to be able to meet the parents of children during an admission to hospital with acute bronchiolitis, to gauge their	

ADDITIONAL FILE 2

Table 2. Full study characteristics of included studies.

† Denotes where excerpts are taken verbatim from study.

Study	Country of conduct	Title	Multi-centre/ single centre	Sample Size	Field/ Topic	Ethnic/ Minority populations	Number of patients/ community representatives engaged in research	Methods of Engagement†			
								Development of research question	*Selecting outcome*	*Other activities*	*Dissemination and Implementation of results*
										perspective on our proposed trial at a similar time point as that in which we would be engaging with parents of children enrolled to the study. Parents were more positive than we had expected	

ADDITIONAL FILE 2

Table 2. Full study characteristics of included studies.

† Denotes where excerpts are taken verbatim from study.

**Study**	**Country of conduct**	**Title**	**Multi-centre/ single centre**	**Sample Size**	**Field/ Topic**	**Ethnic/ Minority populations**	**Number of patients/ community representatives engaged in research**	**Methods of Engagement†**			
								Development of research question	*Selecting outcome*	*Other activities*	*Dissemination and Implementation of results*
										and the discussions gave valuable guidance for study exclusions, parent information sheets and appropriate use of language when conveying potential risks and benefits of the study.	

ADDITIONAL FILE 2

Table 2. Full study characteristics of included studies.

† Denotes where excerpts are taken verbatim from study.

**Study**	**Country of conduct**	**Title**	**Multi-centre/ single centre**	**Sample Size**	**Field/ Topic**	**Ethnic/ Minority populations**	**Number of patients/ community representatives engaged in research**	**Methods of Engagement†**			
								Development of research question	*Selecting outcome*	*Other activities*	*Dissemination and Implementation of results*
Zgibor, J.C., 2016 [38]	USA	Partnership Building and Implementation of an Integrated Healthy-Aging Program	Multiple sites	462	Health aging	Blacks, other	NR	Not reported	Not reported	Both the AF and CAPH-PRC recruited instructors, integrated the content of the programs, and shared training costs. The CAPH-PRC was responsible for new site and participant	Not reported

ADDITIONAL FILE 2

Table 2. Full study characteristics of included studies.

† Denotes where excerpts are taken verbatim from study.

**Study**	**Country of conduct**	**Title**	**Multi-centre/ single centre**	**Sample Size**	**Field/ Topic**	**Ethnic/ Minority populations**	**Number of patients/ community representatives engaged in research**	**Methods of Engagement†**			
								Development of research question	*Selecting outcome*	*Other activities*	*Dissemination and Implementation of results*
										recruitment and research-related tasks including data collection, data entry, and analysis, and the AF was responsible for facilitating engagement of existing sites and	

ADDITIONAL FILE 2

Table 2. Full study characteristics of included studies.

† Denotes where excerpts are taken verbatim from study.

**Study**	**Country of conduct**	**Title**	**Multi-centre/ single centre**	**Sample Size**	**Field/ Topic**	**Ethnic/ Minority populations**	**Number of patients/ community representatives engaged in research**	**Methods of Engagement†**			
								Development of research question	*Selecting outcome*	*Other activities*	*Dissemination and Implementation of results*
										training new instructors. A Research Advisory Board was formed consisting of two representatives from the AF and three representatives from CAPH-PRC. Bi-weekly meetings were held	

ADDITIONAL FILE 2

Table 2. Full study characteristics of included studies.

† Denotes where excerpts are taken verbatim from study.

Study	Country of conduct	Title	Multi-centre/ single centre	Sample Size	Field/ Topic	Ethnic/ Minority populations	Number of patients/ community representatives engaged in research	Methods of Engagement†			
								Development of research question	*Selecting outcome*	*Other activities*	*Dissemination and Implementation of results*
										with additional contact made as needed in order to address any program challenges. Although the study design and selection of measures was driven by the researchers, the AF had	

ADDITIONAL FILE 2

Table 2. Full study characteristics of included studies.

† Denotes where excerpts are taken verbatim from study.

**Study**	**Country of conduct**	**Title**	**Multi-centre/ single centre**	**Sample Size**	**Field/ Topic**	**Ethnic/ Minority populations**	**Number of patients/ community representatives engaged in research**	**Methods of Engagement†**			
								Development of research question	*Selecting outcome*	*Other activities*	*Dissemination and Implementation of results*
										considerable input into training and implementation. The CAPH-PRC provided all recruitment materials, instructor manuals, participant materials, training on human subject research, and a small	

ADDITIONAL FILE 2

Table 2. Full study characteristics of included studies.

† Denotes where excerpts are taken verbatim from study.

Study	Country of conduct	Title	Multi-centre/ single centre	Sample Size	Field/ Topic	Ethnic/ Minority populations	Number of patients/ community representatives engaged in research	Methods of Engagement†			
								Development of research question	*Selecting outcome*	*Other activities*	*Dissemination and Implementation of results*
										stipend for instructors. A representative from the AF also sat on the Data Safety Monitoring Committee for the study.	
Man, M.S., 2015 [25]	UK	Improving recruitment to a study of telehealth management for long-term conditions	Multiple sites	2 trials embedded in study: Healthlines CVD (n	Patient information aids	Not reported	2	Not reported	Not reported	Study does not state how patients were engaged. However	Not reported

ADDITIONAL FILE 2

Table 2. Full study characteristics of included studies.

† Denotes where excerpts are taken verbatim from study.

**Study**	**Country of conduct**	**Title**	**Multi-centre/ single centre**	**Sample Size**	**Field/ Topic**	**Ethnic/ Minority populations**	**Number of patients/ community representatives engaged in research**	**Methods of Engagement†**			
								Development of research question	*Selecting outcome*	*Other activities*	*Dissemination and Implementation of results*
		in primary care: two embedded, randomised controlled trials of optimised patient information materials		= 671); Healthlines Depression (n = 1364)						they do state "Judith Hogg and Ailsa Donnelly from PRIMER (the Primary Care Research in Manchester Engagement Resource) who contributed patient and public	

ADDITIONAL FILE 2

Table 2. Full study characteristics of included studies.

† Denotes where excerpts are taken verbatim from study.

Study	Country of conduct	Title	Multi-centre/ single centre	Sample Size	Field/ Topic	Ethnic/ Minority populations	Number of patients/ community representatives engaged in research	Methods of Engagement†			
								Development of research question	*Selecting outcome*	*Other activities*	*Dissemination and Implementation of results*
										involvement to the work of the MRC-START Study team" (In acknowledgments)<br><br>Developing the recruitment intervention: "In MRC-START, a process involving	

ADDITIONAL FILE 2

Table 2. Full study characteristics of included studies.

† Denotes where excerpts are taken verbatim from study.

**Study**	**Country of conduct**	**Title**	**Multi-centre/ single centre**	**Sample Size**	**Field/ Topic**	**Ethnic/ Minority populations**	**Number of patients/ community representatives engaged in research**	**Methods of Engagement†**			
								Development of research question	*Selecting outcome*	*Other activities*	*Dissemination and Implementation of results*
										consumer feedback, expertise in writing for patients and graphic design is used to produce an optimised version of the patient information materials (patient information sheet and covering letter) for	

ADDITIONAL FILE 2

Table 2. Full study characteristics of included studies.

† Denotes where excerpts are taken verbatim from study.

Study	Country of conduct	Title	Multi-centre/ single centre	Sample Size	Field/ Topic	Ethnic/ Minority populations	Number of patients/ community representatives engaged in research	Methods of Engagement†			
								Development of research question	*Selecting outcome*	*Other activities*	*Dissemination and Implementation of results*
										each host trial"	
Bauermeister, J.A., 2015 [30]	USA	Acceptability and Preliminary Efficacy of a Tailored Online HIV/STI Testing Intervention for Young Men who have Sex with Men: The Get	Multiple sites	130	HIV/STI Testing Intervention	The CAB (community advisory board) were also diverse with regards to race/ethnicity (three Whites, two Latinos, and two African American). The YAB:	The community advisory board includes consisted of seven community organization. The youth advisory board consisted of eight members	Not reported	Not reported	"We thank our CAB and YAB for their contributions during the development and implementation of the intervention. "All study procedures were approved	Not reported

ADDITIONAL FILE 2

Table 2. Full study characteristics of included studies.

† Denotes where excerpts are taken verbatim from study.

**Study**	**Country of conduct**	**Title**	**Multi-centre/ single centre**	**Sample Size**	**Field/ Topic**	**Ethnic/ Minority populations**	**Number of patients/ community representatives engaged in research**	**Methods of Engagement†**			
								Development of research question	*Selecting outcome*	*Other activities*	*Dissemination and Implementation of results*
		Connected! Program				four African American, two White, one Middle Eastern, and one Mixed Race (African American/ White).				by Community Advisory Board (CAB) and Youth Advisory Board (YAB).<br><br>The CAB met with the Get Connected! research team four times, providing constructive	

ADDITIONAL FILE 2

Table 2. Full study characteristics of included studies.

† Denotes where excerpts are taken verbatim from study.

Study	Country of conduct	Title	Multi-centre/ single centre	Sample Size	Field/ Topic	Ethnic/ Minority populations	Number of patients/ community representatives engaged in research	Methods of Engagement†			
								Development of research question	*Selecting outcome*	*Other activities*	*Dissemination and Implementation of results*
										feedback on most aspects of the study's progression, including the development of the provider checklist, which was used to evaluate the HIV/STI testing sites, and the online	

ADDITIONAL FILE 2

Table 2. Full study characteristics of included studies.

† Denotes where excerpts are taken verbatim from study.

Study	Country of conduct	Title	Multi-centre/ single centre	Sample Size	Field/ Topic	Ethnic/ Minority populations	Number of patients/ community representatives engaged in research	Methods of Engagement†			
								Development of research question	*Selecting outcome*	*Other activities*	*Dissemination and Implementation of results*
										tailored website. They were invaluable in helping to assemble critical questions for study participants to ask providers upon getting tested as well as developing the "STI	

ADDITIONAL FILE 2

Table 2. Full study characteristics of included studies.

† Denotes where excerpts are taken verbatim from study.

**Study**	**Country of conduct**	**Title**	**Multi-centre/ single centre**	**Sample Size**	**Field/ Topic**	**Ethnic/ Minority populations**	**Number of patients/ community representatives engaged in research**	**Methods of Engagement†**			
								Development of research question	*Selecting outcome*	*Other activities*	*Dissemination and Implementation of results*
										facts'' portion of the website.<br><br>The YAB met with the Get Connected! research team six times throughout the study, providing critical feedback at each stage of the	

ADDITIONAL FILE 2

Table 2. Full study characteristics of included studies.

† Denotes where excerpts are taken verbatim from study.

**Study**	**Country of conduct**	**Title**	**Multi-centre/ single centre**	**Sample Size**	**Field/ Topic**	**Ethnic/ Minority populations**	**Number of patients/ community representatives engaged in research**	**Methods of Engagement†**			
								Development of research question	*Selecting outcome*	*Other activities*	*Dissemination and Implementation of results*
										project. As members of the study's target population, their insights were crucial to the success of the study. In collaboration with a graphic designer, the YAB members designed	

ADDITIONAL FILE 2

Table 2. Full study characteristics of included studies.

† Denotes where excerpts are taken verbatim from study.

**Study**	**Country of conduct**	**Title**	**Multi-centre/ single centre**	**Sample Size**	**Field/ Topic**	**Ethnic/ Minority populations**	**Number of patients/ community representatives engaged in research**	**Methods of Engagement†**			
								Development of research question	*Selecting outcome*	*Other activities*	*Dissemination and Implementation of results*
										the logo and recruitment materials (e.g., palm cards) for Get Connected! They critiqued each iteration of the website design and gave extensive feedback on both the content presented	

ADDITIONAL FILE 2

Table 2. Full study characteristics of included studies.

† Denotes where excerpts are taken verbatim from study.

**Study**	**Country of conduct**	**Title**	**Multi-centre/ single centre**	**Sample Size**	**Field/ Topic**	**Ethnic/ Minority populations**	**Number of patients/ community representatives engaged in research**	**Methods of Engagement†**			
								Development of research question	*Selecting outcome*	*Other activities*	*Dissemination and Implementation of results*
										on each page of the site and the language used in the survey items. YAB members also assisted with study participant recruitment.<br><br>As the content for the intervention	

ADDITIONAL FILE 2

Table 2. Full study characteristics of included studies.

† Denotes where excerpts are taken verbatim from study.

**Study**	**Country of conduct**	**Title**	**Multi-centre/ single centre**	**Sample Size**	**Field/ Topic**	**Ethnic/ Minority populations**	**Number of patients/ community representatives engaged in research**	**Methods of Engagement†**			
								Development of research question	*Selecting outcome*	*Other activities*	*Dissemination and Implementation of results*
										and control conditions (including imagery and messaging) was developed, focus groups consisting of YAB and CAB members evaluated this content for its acceptability and relevance	

ADDITIONAL FILE 2

Table 2. Full study characteristics of included studies.

† Denotes where excerpts are taken verbatim from study.

Study	Country of conduct	Title	Multi-centre/ single centre	Sample Size	Field/ Topic	Ethnic/ Minority populations	Number of patients/ community representatives engaged in research	Methods of Engagement†			
								Development of research question	*Selecting outcome*	*Other activities*	*Dissemination and Implementation of results*
										to the target population.	
Huppelschoten, A, G. 2015 [39]	Netherlands	Improving patient-centredness in partnership with female patients: a cluster RCT in fertility care	Multiple sites	730	Patient-centered fertility care	Not reported	Not reported	Not reported	audit and feedback procedure and in the outcome measurements	"The Dutch Patient Association 'Freya' was involved in the design of the intervention. "<br><br>"This randomized study on the improveme	Not reported

ADDITIONAL FILE 2

Table 2. Full study characteristics of included studies.

† Denotes where excerpts are taken verbatim from study.

Study	Country of conduct	Title	Multi-centre/ single centre	Sample Size	Field/ Topic	Ethnic/ Minority populations	Number of patients/ community representatives engaged in research	Methods of Engagement†			
								Development of research question	*Selecting outcome*	*Other activities*	*Dissemination and Implementation of results*
										nt of patient-centredness was done in full partnership with patients. They were involved in the design of the study, in the development of the questionnaires, in the	

ADDITIONAL FILE 2

Table 2. Full study characteristics of included studies.

[†] Denotes where excerpts are taken verbatim from study.

**Study**	**Country of conduct**	**Title**	**Multi-centre/ single centre**	**Sample Size**	**Field/ Topic**	**Ethnic/ Minority populations**	**Number of patients/ community representatives engaged in research**	**Methods of Engagement[†]**			
								Development of research question	*Selecting outcome*	*Other activities*	*Dissemination and Implementation of results*
										audit and feedback procedure and in the outcome measurements. Moreover, patients played an important role in the execution of the intervention, as former patients of the participating clinics (i.e.	

ADDITIONAL FILE 2

Table 2. Full study characteristics of included studies.

† Denotes where excerpts are taken verbatim from study.

**Study**	**Country of conduct**	**Title**	**Multi-centre/ single centre**	**Sample Size**	**Field/ Topic**	**Ethnic/ Minority populations**	**Number of patients/ community representatives engaged in research**	**Methods of Engagement†**			
								Development of research question	*Selecting outcome*	*Other activities*	*Dissemination and Implementation of results*
										both male and female patients) took part in the EOVs as consultants of the team."	
Tully, M., 2015, [26]	USA	Implementation of an Adjunct Strategy to Reduce Blood Pressure in Blacks with Uncontrolled	Multiple sites	83	Hypertension	African-Americans	Not reported	Not reported	Not reported	"The community health worker (CHW) then contacted the patients again	Not reported

ADDITIONAL FILE 2

Table 2. Full study characteristics of included studies.

† Denotes where excerpts are taken verbatim from study.

**Study**	**Country of conduct**	**Title**	**Multi-centre/ single centre**	**Sample Size**	**Field/ Topic**	**Ethnic/ Minority populations**	**Number of patients/ community representatives engaged in research**	**Methods of Engagement†**			
								Development of research question	*Selecting outcome*	*Other activities*	*Dissemination and Implementation of results*
		Hypertension: a Pilot Project								to field questions about study participation and offer convenient meeting times. She followed up with reminder calls, sent bus tickets and remained available for questions.<br><br>"The CHW, a black	

ADDITIONAL FILE 2

Table 2. Full study characteristics of included studies.

† Denotes where excerpts are taken verbatim from study.

Study	Country of conduct	Title	Multi-centre/ single centre	Sample Size	Field/ Topic	Ethnic/ Minority populations	Number of patients/ community representatives engaged in research	Methods of Engagement†			
								Development of research question	*Selecting outcome*	*Other activities*	*Dissemination and Implementation of results*
										woman, functioned as a lay leader for the intervention. This was a full time position with fringe benefits. She had no other responsibilities. The individual in this position did not have a college	

ADDITIONAL FILE 2

Table 2. Full study characteristics of included studies.

† Denotes where excerpts are taken verbatim from study.

Study	Country of conduct	Title	Multi-centre/ single centre	Sample Size	Field/ Topic	Ethnic/ Minority populations	Number of patients/ community representatives engaged in research	Methods of Engagement†			
								Development of research question	*Selecting outcome*	*Other activities*	*Dissemination and Implementation of results*
										degree but had a long history in primary education before becoming an AHEC certified Community Health Worker. She was also certified through the Living Well with Chronic Diseases	

ADDITIONAL FILE 2

Table 2. Full study characteristics of included studies.

† Denotes where excerpts are taken verbatim from study.

Study	Country of conduct	Title	Multi-centre/ single centre	Sample Size	Field/ Topic	Ethnic/ Minority populations	Number of patients/ community representatives engaged in research	Methods of Engagement†			
								Development of research question	*Selecting outcome*	*Other activities*	*Dissemination and Implementation of results*
										program (10). During the intervention, she reinforced individual patient action plans with weekly telephone calls to each participant. She assessed goals, reminded patients about	

ADDITIONAL FILE 2

Table 2. Full study characteristics of included studies.

† Denotes where excerpts are taken verbatim from study.

Study	Country of conduct	Title	Multi-centre/ single centre	Sample Size	Field/ Topic	Ethnic/ Minority populations	Number of patients/ community representatives engaged in research	Methods of Engagement†			
								Development of research question	*Selecting outcome*	*Other activities*	*Dissemination and Implementation of results*
										scheduled group sessions, and provided encouragement. She served as a confidant, a source of information, and facilitated communication between patients, social workers, and	

ADDITIONAL FILE 2

Table 2. Full study characteristics of included studies.

† Denotes where excerpts are taken verbatim from study.

**Study**	**Country of conduct**	**Title**	**Multi-centre/ single centre**	**Sample Size**	**Field/ Topic**	**Ethnic/ Minority populations**	**Number of patients/ community representatives engaged in research**	**Methods of Engagement†**			
								Development of research question	*Selecting outcome*	*Other activities*	*Dissemination and Implementation of results*
										healthcare providers."<br><br>"A Community Advisory Board was established to inform the recruitment process, to review the study's progress and barriers, and to provide guidance in	

ADDITIONAL FILE 2

Table 2. Full study characteristics of included studies.

† Denotes where excerpts are taken verbatim from study.

Study	Country of conduct	Title	Multi-centre/ single centre	Sample Size	Field/ Topic	Ethnic/ Minority populations	Number of patients/ community representatives engaged in research	Methods of Engagement†			
								Development of research question	*Selecting outcome*	*Other activities*	*Dissemination and Implementation of results*
										selecting and developing culturally sensitive educational materials and strategies related to health promotion and hypertension. The Board was comprised of representatives from	

ADDITIONAL FILE 2

Table 2. Full study characteristics of included studies.

† Denotes where excerpts are taken verbatim from study.

**Study**	**Country of conduct**	**Title**	**Multi-centre/ single centre**	**Sample Size**	**Field/ Topic**	**Ethnic/ Minority populations**	**Number of patients/ community representatives engaged in research**	**Methods of Engagement†**			
								Development of research question	*Selecting outcome*	*Other activities*	*Dissemination and Implementation of results*
										nonprofit organizations with a stake in Milwaukee's at-risk black community. Membership included one representative from each of the following: the American Heart Association, a	

ADDITIONAL FILE 2

Table 2. Full study characteristics of included studies.

† Denotes where excerpts are taken verbatim from study.

**Study**	**Country of conduct**	**Title**	**Multi-centre/ single centre**	**Sample Size**	**Field/ Topic**	**Ethnic/ Minority populations**	**Number of patients/ community representatives engaged in research**	**Methods of Engagement†**			
								Development of research question	*Selecting outcome*	*Other activities*	*Dissemination and Implementation of results*
										neighborhood Health Alliance, the Wisconsin Primary Health Care Association, and a community representative who is a locally recognized chef and who personally has	

ADDITIONAL FILE 2

Table 2. Full study characteristics of included studies.

† Denotes where excerpts are taken verbatim from study.

**Study**	**Country of conduct**	**Title**	**Multi-centre/ single centre**	**Sample Size**	**Field/ Topic**	**Ethnic/ Minority populations**	**Number of patients/ community representatives engaged in research**	**Methods of Engagement†**			
								Development of research question	*Selecting outcome*	*Other activities*	*Dissemination and Implementation of results*
										hypertension. The Board met a total of 11 times during the course of the study, more frequently in the planning phases and subsequently quarterly."	
Ormerod, A.D., 2015 [40]	UK	Comparison of the two most commonly	Multiple sites	121	Pyoderma gangrenosum	Not reported	Not reported	During the feasibility stage,	During the feasibility	Patients were involved in the design	Not reported

ADDITIONAL FILE 2

Table 2. Full study characteristics of included studies.

† Denotes where excerpts are taken verbatim from study.

**Study**	**Country of conduct**	**Title**	**Multi-centre/ single centre**	**Sample Size**	**Field/ Topic**	**Ethnic/ Minority populations**	**Number of patients/ community representatives engaged in research**	**Methods of Engagement†**			
								Development of research question	*Selecting outcome*	*Other activities*	*Dissemination and Implementation of results*
		used treatments for pyoderma gangrenosum: results of the STOP GAP randomised controlled trial						priority of the research question, choice of outcome measures, and methods of recruitment were informed by discussions with patients through	stage, priority of the research question, choice of outcome measures, and methods of recruitment were	and conduct of this research. During the feasibility stage, priority of the research question, choice of outcome measures, and methods of recruitment were informed by	

ADDITIONAL FILE 2

Table 2. Full study characteristics of included studies.

† Denotes where excerpts are taken verbatim from study.

**Study**	**Country of conduct**	**Title**	**Multi-centre/ single centre**	**Sample Size**	**Field/ Topic**	**Ethnic/ Minority populations**	**Number of patients/ community representatives engaged in research**	**Methods of Engagement†**			
								Development of research question	*Selecting outcome*	*Other activities*	*Dissemination and Implementation of results*
								a focus group session and two structured interviews.	informed by discussions with patients through a focus group session and two structured interviews.	discussions with patients through a focus group session and two structured interviews. During the trial, a patient joined the independent trial steering committee. Members of the UK Dermatolog	

ADDITIONAL FILE 2

Table 2. Full study characteristics of included studies.

† Denotes where excerpts are taken verbatim from study.

Study	Country of conduct	Title	Multi-centre/ single centre	Sample Size	Field/ Topic	Ethnic/ Minority populations	Number of patients/ community representatives engaged in research	Methods of Engagement†			
								Development of research question	*Selecting outcome*	*Other activities*	*Dissemination and Implementation of results*
										y Clinical Trials Network also identified this research as being a priority area for clinicians treating patients with pyoderma gangrenosum. Once the trial has been published,	

ADDITIONAL FILE 2

Table 2. Full study characteristics of included studies.

† Denotes where excerpts are taken verbatim from study.

**Study**	**Country of conduct**	**Title**	**Multi-centre/ single centre**	**Sample Size**	**Field/ Topic**	**Ethnic/ Minority populations**	**Number of patients/ community representatives engaged in research**	**Methods of Engagement†**			
								Development of research question	*Selecting outcome*	*Other activities*	*Dissemination and Implementation of results*
										participants will be informed of the results through a dedicated website (www.stopgaptrial.co.uk) and will be sent details of the results in a study newsletter suitable for a non-specialist audience	

ADDITIONAL FILE 2

Table 2. Full study characteristics of included studies.

† Denotes where excerpts are taken verbatim from study.

**Study**	**Country of conduct**	**Title**	**Multi-centre/ single centre**	**Sample Size**	**Field/ Topic**	**Ethnic/ Minority populations**	**Number of patients/ community representatives engaged in research**	**Methods of Engagement†**			
								Development of research question	*Selecting outcome*	*Other activities*	*Dissemination and Implementation of results*
Goodacre., 2015 [27]	UK	The PAndemic INfluenza Triage in the Emergency Department (PAINTED) pilot cohort study	Multiple sites	165	Influenza	Not reported	Not reported	Enid Hirst has agreed to be the patient/public representative for the project and has reviewed the proposal. She acted as patient and public represen	Not reported	Enid's role will include the following: 2. reviewing the poster and information leaflet 3. patient/public representation on the Steering Committee 4. lay input into reporting	Enid's role include lay input into reporting and dissemination of findings

ADDITIONAL FILE 2

Table 2. Full study characteristics of included studies.

† Denotes where excerpts are taken verbatim from study.

**Study**	**Country of conduct**	**Title**	**Multi-centre/ single centre**	**Sample Size**	**Field/ Topic**	**Ethnic/ Minority populations**	**Number of patients/ community representatives engaged in research**	**Methods of Engagement†**			
								Development of research question	*Selecting outcome*	*Other activities*	*Dissemination and Implementation of results*
								tative for our project in the 2009 pandemic and was an independent member of the study Steering Committee. Enid is a founder member		and dissemination of findings<br>5. liaison between the project and the Sheffield Emergency Care Forum.<br><br>Lynn Winspear, Enid Hirst and other members of SECF also	

ADDITIONAL FILE 2

Table 2. Full study characteristics of included studies.

† Denotes where excerpts are taken verbatim from study.

**Study**	**Country of conduct**	**Title**	**Multi-centre/ single centre**	**Sample Size**	**Field/ Topic**	**Ethnic/ Minority populations**	**Number of patients/ community representatives engaged in research**	**Methods of Engagement†**			
								Development of research question	*Selecting outcome*	*Other activities*	*Dissemination and Implementation of results*
								of the Sheffield Emergency Care Forum. This is a patient and public representative group with a specific interest in emergency care research		provided specific input into the project's lay summary and patient leaflets	

ADDITIONAL FILE 2

Table 2. Full study characteristics of included studies.

† Denotes where excerpts are taken verbatim from study.

**Study**	**Country of conduct**	**Title**	**Multi-centre/ single centre**	**Sample Size**	**Field/ Topic**	**Ethnic/ Minority populations**	**Number of patients/ community representatives engaged in research**	**Methods of Engagement†**			
								Development of research question	*Selecting outcome*	*Other activities*	*Dissemination and Implementation of results*
								. The Forum has reviewed this proposal and provided feedback. Enid will continue to provide a link between the project and the Forum.			

ADDITIONAL FILE 2

Table 2. Full study characteristics of included studies.

† Denotes where excerpts are taken verbatim from study.

**Study**	**Country of conduct**	**Title**	**Multi-centre/ single centre**	**Sample Size**	**Field/ Topic**	**Ethnic/ Minority populations**	**Number of patients/ community representatives engaged in research**	**Methods of Engagement†**			
								Development of research question	*Selecting outcome*	*Other activities*	*Dissemination and Implementation of results*
								Her role will include the following: 1. reviewing the protocol and specifically advising on ethical issues and arrangements			

ADDITIONAL FILE 2

Table 2. Full study characteristics of included studies.

† Denotes where excerpts are taken verbatim from study.

Study	Country of conduct	Title	Multi-centre/ single centre	Sample Size	Field/ Topic	Ethnic/ Minority populations	Number of patients/ community representatives engaged in research	Methods of Engagement†			
								Development of research question	*Selecting outcome*	*Other activities*	*Dissemination and Implementation of results*
								for data protection and confidentiality			
Mohatt, G.V., 2014 [28]	USA	Feasibility of a Community Intervention for the Prevention of Suicide and Alcohol Abuse with Yup'ik Alaska Native Youth: The Elluam	Multiple sites	ET study- 61 youth<br>YA study- 53 youth	Prevention of Suicide and Alcohol Abuse	Yup'ik ethnicity	Not reported	Not reported	Not reported	Planning, community development work, and development of prevention activity modules that would constitute the future	Not reported

ADDITIONAL FILE 2

Table 2. Full study characteristics of included studies.

† Denotes where excerpts are taken verbatim from study.

Study	Country of conduct	Title	Multi-centre/ single centre	Sample Size	Field/ Topic	Ethnic/ Minority populations	Number of patients/ community representatives engaged in research	Methods of Engagement†			
								Development of research question	*Selecting outcome*	*Other activities*	*Dissemination and Implementation of results*
		Tungiinun and Yupiucimta Asvairtuum allerkaa Studies								intervention as implemented was conducted by community members with the assistance of local and university project staff over a one (YA) or two (ET) year time period prior to any module	

ADDITIONAL FILE 2

Table 2. Full study characteristics of included studies.

† Denotes where excerpts are taken verbatim from study.

**Study**	**Country of conduct**	**Title**	**Multi-centre/ single centre**	**Sample Size**	**Field/ Topic**	**Ethnic/ Minority populations**	**Number of patients/ community representatives engaged in research**	**Methods of Engagement†**			
								Development of research question	*Selecting outcome*	*Other activities*	*Dissemination and Implementation of results*
										delivery in the intervention activities.<br><br>Community planning groups developed the interventions with representation from youth, parents, community leadership, Elders, and	

ADDITIONAL FILE 2

Table 2. Full study characteristics of included studies.

† Denotes where excerpts are taken verbatim from study.

Study	Country of conduct	Title	Multi-centre/ single centre	Sample Size	Field/ Topic	Ethnic/ Minority populations	Number of patients/ community representatives engaged in research	Methods of Engagement†			
								Development of research question	*Selecting outcome*	*Other activities*	*Dissemination and Implementation of results*
										university researchers through a process described in Rasmus et al. (this issue). This work, in the ET community, resulted in the Qungasvik (toolbox; Alakanuk Community Planning Group et al., 2008).	

ADDITIONAL FILE 2

Table 2. Full study characteristics of included studies.

† Denotes where excerpts are taken verbatim from study.

Study	Country of conduct	Title	Multi-centre/ single centre	Sample Size	Field/ Topic	Ethnic/ Minority populations	Number of patients/ community representatives engaged in research	Methods of Engagement†			
								Development of research question	*Selecting outcome*	*Other activities*	*Dissemination and Implementation of results*
										Rather than offering a prescriptive manual of specific intervention activities, the Qungasvik was developed as a process manual for intervention. It provides a flexible format, selection from a	

ADDITIONAL FILE 2

Table 2. Full study characteristics of included studies.

† Denotes where excerpts are taken verbatim from study.

Study	Country of conduct	Title	Multi-centre/ single centre	Sample Size	Field/ Topic	Ethnic/ Minority populations	Number of patients/ community representatives engaged in research	Methods of Engagement†			
								Development of research question	*Selecting outcome*	*Other activities*	*Dissemination and Implementation of results*
										range of activities at different levels (individual, family, community), and basic outlines for prevention activities, all situated within a community development framework.	

ADDITIONAL FILE 2

Table 2. Full study characteristics of included studies.

† Denotes where excerpts are taken verbatim from study.

Study	Country of conduct	Title	Multi-centre/ single centre	Sample Size	Field/ Topic	Ethnic/ Minority populations	Number of patients/ community representatives engaged in research	Methods of Engagement†			
								Development of research question	*Selecting outcome*	*Other activities*	*Dissemination and Implementation of results*
Koladooz, F., 2014 [29]	Canada	Impact of the Healthy Foods North nutrition intervention program on Inuit and Inuvialuit food consumption and preparation methods in Canadian Arctic communities	Multiple sites	332	Nutrition	Canadian Indigenous populations	Not reported	Not reported	Not reported	Community participatory research [24] was used to identify the themes of the interventions and have previously been described in detail [13]"<br><br>Ref[13]: "On the basis of dietary	Not reported

ADDITIONAL FILE 2

Table 2. Full study characteristics of included studies.

† Denotes where excerpts are taken verbatim from study.

**Study**	**Country of conduct**	**Title**	**Multi-centre/ single centre**	**Sample Size**	**Field/ Topic**	**Ethnic/ Minority populations**	**Number of patients/ community representatives engaged in research**	**Methods of Engagement†**			
								Development of research question	*Selecting outcome*	*Other activities*	*Dissemination and Implementation of results*
										intake data (Sharma et al., 2009, 2010a), formative research (Gittelsohn et al., 2010) and using a community participatory process, a novel, integrated, multi-institutional chronic disease	

ADDITIONAL FILE 2

Table 2. Full study characteristics of included studies.

† Denotes where excerpts are taken verbatim from study.

Study	Country of conduct	Title	Multi-centre/ single centre	Sample Size	Field/ Topic	Ethnic/ Minority populations	Number of patients/ community representatives engaged in research	Methods of Engagement†			
								Development of research question	*Selecting outcome*	*Other activities*	*Dissemination and Implementation of results*
										prevention programme called Healthy Foods North (HFN) was developed for Inuit and Inuvialuit in the Canadian Arctic"	
O'Callaghan, P., 2014 [32]	UK	A pilot study of a family focused, psychosocial	Multiple sites	159	Post-traumatic stress symptoms	Not reported	Not reported	Not reported	Not reported	The study protocol was approval by the ethical	Not reported

ADDITIONAL FILE 2

Table 2. Full study characteristics of included studies.

† Denotes where excerpts are taken verbatim from study.

Study	Country of conduct	Title	Multi-centre/ single centre	Sample Size	Field/ Topic	Ethnic/ Minority populations	Number of patients/ community representatives engaged in research	Methods of Engagement†			
								Development of research question	*Selecting outcome*	*Other activities*	*Dissemination and Implementation of results*
		intervention with war-exposed youth at risk of attack and abduction in north-eastern Democratic Republic of Congo								review board in the lead researcher's university and then by a 'Community Advisory Board', headed by a community pastor with a Masters in trauma interventions with youth victims of community	

ADDITIONAL FILE 2

Table 2. Full study characteristics of included studies.

† Denotes where excerpts are taken verbatim from study.

**Study**	**Country of conduct**	**Title**	**Multi-centre/ single centre**	**Sample Size**	**Field/ Topic**	**Ethnic/ Minority populations**	**Number of patients/ community representatives engaged in research**	**Methods of Engagement†**			
								Development of research question	*Selecting outcome*	*Other activities*	*Dissemination and Implementation of results*
										violence. This Board was set up to advise on the appropriateness of interview questions, assist in drafting and piloting questionnaires and assess the psychological distress of children and young people in	

ADDITIONAL FILE 2

Table 2. Full study characteristics of included studies.

† Denotes where excerpts are taken verbatim from study.

Study	Country of conduct	Title	Multi-centre/ single centre	Sample Size	Field/ Topic	Ethnic/ Minority populations	Number of patients/ community representatives engaged in research	Methods of Engagement†			
								Development of research question	*Selecting outcome*	*Other activities*	*Dissemination and Implementation of results*
										the study. Prior to enrolment, the protocol was submitted to the UNHCR Protection Cluster for review by UN and NGO project managers with experience delivering psycho-	

ADDITIONAL FILE 2

Table 2. Full study characteristics of included studies.

† Denotes where excerpts are taken verbatim from study.

Study	Country of conduct	Title	Multi-centre/ single centre	Sample Size	Field/ Topic	Ethnic/ Minority populations	Number of patients/ community representatives engaged in research	Methods of Engagement†			
								Development of research question	*Selecting outcome*	*Other activities*	*Dissemination and Implementation of results*
										social interventions.	
Bowrey, D.J., 2014 [22]	UK	A randomised controlled trial of six weeks of home enteral nutrition versus standard care after oesophagectomy or total gastrectomy for cancer: report on a	Single site	54	Home enteral nutrition after esophagectomy or total gastrectomy for cancer	Not reported	Not reported	Not reported	Not reported	No specific details about how patients were involved in the full text. However, authors state in protocol "A trial management group has been setup	Not reported

ADDITIONAL FILE 2

Table 2. Full study characteristics of included studies.

† Denotes where excerpts are taken verbatim from study.

Study	Country of conduct	Title	Multi-centre/ single centre	Sample Size	Field/ Topic	Ethnic/ Minority populations	Number of patients/ community representatives engaged in research	Methods of Engagement†			
								Development of research question	*Selecting outcome*	*Other activities*	*Dissemination and Implementation of results*
		pilot and feasibility study								including the investigators, representatives from the Leicester Clinical Trials Unit, and two former patients (one with their partner). This group will meet every six	

ADDITIONAL FILE 2

Table 2. Full study characteristics of included studies.

† Denotes where excerpts are taken verbatim from study.

**Study**	**Country of conduct**	**Title**	**Multi-centre/ single centre**	**Sample Size**	**Field/ Topic**	**Ethnic/ Minority populations**	**Number of patients/ community representatives engaged in research**	**Methods of Engagement†**			
								Development of research question	*Selecting outcome*	*Other activities*	*Dissemination and Implementation of results*
										months for the duration of the study to review study progress"	
Littlewood, E., 2015 [41]	UK	A randomised controlled trial of computerised cognitive behaviour therapy for the treatment of depression in primary care: the Randomise	Multiple sites	691	Depression Treatment	Mixed (white and black African), Mixed (white and black Carribbean), Any other mixed background, Asian or Asian	Not reported	Not reported	Not reported	The REEACT trial received patient and public participation input at the design, conduct, analysis and	Not reported

ADDITIONAL FILE 2

Table 2. Full study characteristics of included studies.

† Denotes where excerpts are taken verbatim from study.

Study	Country of conduct	Title	Multi-centre/ single centre	Sample Size	Field/ Topic	Ethnic/ Minority populations	Number of patients/ community representatives engaged in research	Methods of Engagement†			
								Development of research question	*Selecting outcome*	*Other activities*	*Dissemination and Implementation of results*
		d Evaluation of the Effectiveness and Acceptability of Computerised Therapy (REEACT) trial				british-indian, Asian or Asian British-Pakistani, Chinese, Other				interpretation stages. We received input from those with lived experience of depression in the design of all research materials. The REEACT trial received oversight via	

ADDITIONAL FILE 2

Table 2. Full study characteristics of included studies.

† Denotes where excerpts are taken verbatim from study.

**Study**	**Country of conduct**	**Title**	**Multi-centre/ single centre**	**Sample Size**	**Field/ Topic**	**Ethnic/ Minority populations**	**Number of patients/ community representatives engaged in research**	**Methods of Engagement†**			
								Development of research question	*Selecting outcome*	*Other activities*	*Dissemination and Implementation of results*
										committee membership from members with lived experience of common mental health problems and a representative of a user-led self-help organisation. A user-led organisatio	

ADDITIONAL FILE 2

Table 2. Full study characteristics of included studies.

† Denotes where excerpts are taken verbatim from study.

**Study**	**Country of conduct**	**Title**	**Multi-centre/ single centre**	**Sample Size**	**Field/ Topic**	**Ethnic/ Minority populations**	**Number of patients/ community representatives engaged in research**	**Methods of Engagement†**			
								Development of research question	*Selecting outcome*	*Other activities*	*Dissemination and Implementation of results*
										n (Anxiety UK and Self-Help Services via its Chief Executive) acted as co-applicant and collaborator on the REEACT trial.	
McMilan, A., 2015. [42]	UK	A multicentre randomised controlled trial and economic evaluation	Multiple sites	278	Sleep Apnoea	Asian, other	2	Not reported	Not reported	Two patient representatives participated in the PREDICT	Frank Govan- raised awareness of the study to

ADDITIONAL FILE 2

Table 2. Full study characteristics of included studies.

† Denotes where excerpts are taken verbatim from study.

Study	Country of conduct	Title	Multi-centre/ single centre	Sample Size	Field/ Topic	Ethnic/ Minority populations	Number of patients/ community representatives engaged in research	Methods of Engagement†			
								Development of research question	*Selecting outcome*	*Other activities*	*Dissemination and Implementation of results*
		of continuous positive airway pressure for the treatment of obstructive sleep apnoea syndrome in older people: PREDICT								management; in particular, Mr Frank Govan from Oxford acted as the patient representative. He attended the TSC meetings and his feedback was very helpful in progressing the trial. For example,	the Sleep Apnoea Trust Association, which in turn, publicised the study with their members. Members of the Welsh Sleep Apnoea Society have also supported the PREDICT

ADDITIONAL FILE 2

Table 2. Full study characteristics of included studies.

† Denotes where excerpts are taken verbatim from study.

**Study**	**Country of conduct**	**Title**	**Multi-centre/ single centre**	**Sample Size**	**Field/ Topic**	**Ethnic/ Minority populations**	**Number of patients/ community representatives engaged in research**	**Methods of Engagement†**			
								Development of research question	*Selecting outcome*	*Other activities*	*Dissemination and Implementation of results*
										he raised awareness of the study to the Sleep Apnoea Trust Association, which in turn, publicised the study with their members. The protocol was discussed with Sleep Apnoea Trust	study by providing publicity for the trial.

ADDITIONAL FILE 2

Table 2. Full study characteristics of included studies.

† Denotes where excerpts are taken verbatim from study.

Study	Country of conduct	Title	Multi-centre/ single centre	Sample Size	Field/ Topic	Ethnic/ Minority populations	Number of patients/ community representatives engaged in research	Methods of Engagement†			
								Development of research question	*Selecting outcome*	*Other activities*	*Dissemination and Implementation of results*
										Association members at their annual meeting in 2012, and we were invited to present the results at their 2014 meeting (www.sleep-apnoea-trust.org/user/image/sm52.pdf). Members of the Welsh Sleep Apnoea	

ADDITIONAL FILE 2

Table 2. Full study characteristics of included studies.

† Denotes where excerpts are taken verbatim from study.

Study	Country of conduct	Title	Multi-centre/ single centre	Sample Size	Field/ Topic	Ethnic/ Minority populations	Number of patients/ community representatives engaged in research	Methods of Engagement†			
								Development of research question	*Selecting outcome*	*Other activities*	*Dissemination and Implementation of results*
										Society have also supported the PREDICT study by providing publicity for the trial. In 2011, Professor Morrell was made an honorary member of the society in recognition of the	

ADDITIONAL FILE 2

Table 2. Full study characteristics of included studies.

† Denotes where excerpts are taken verbatim from study.

Study	Country of conduct	Title	Multi-centre/ single centre	Sample Size	Field/ Topic	Ethnic/ Minority populations	Number of patients/ community representatives engaged in research	Methods of Engagement†			
								Development of research question	*Selecting outcome*	*Other activities*	*Dissemination and Implementation of results*
										research that the team was carrying out (www.welshsas.org). METHODS NIHR Journals Library www.journalslibrary.nihr.ac.uk 16 Mr Govan and other patients regularly discussed	

ADDITIONAL FILE 2

Table 2. Full study characteristics of included studies.

† Denotes where excerpts are taken verbatim from study.

**Study**	**Country of conduct**	**Title**	**Multi-centre/ single centre**	**Sample Size**	**Field/ Topic**	**Ethnic/ Minority populations**	**Number of patients/ community representatives engaged in research**	**Methods of Engagement†**			
								Development of research question	*Selecting outcome*	*Other activities*	*Dissemination and Implementation of results*
										the rigours of participating in research studies with the TMG. These comments have been taken into account in designing subsequent trails. Mr Govern also voted at TSC meetings, and his	

ADDITIONAL FILE 2

Table 2. Full study characteristics of included studies.

† Denotes where excerpts are taken verbatim from study.

**Study**	**Country of conduct**	**Title**	**Multi-centre/ single centre**	**Sample Size**	**Field/ Topic**	**Ethnic/ Minority populations**	**Number of patients/ community representatives engaged in research**	**Methods of Engagement†**			
								Development of research question	*Selecting outcome*	*Other activities*	*Dissemination and Implementation of results*
										independent views were sought when discussing topics such as opening new trial sites, through to trial authorship. The patients who participated in PREDICT from the	

ADDITIONAL FILE 2

Table 2. Full study characteristics of included studies.

† Denotes where excerpts are taken verbatim from study.

Study	Country of conduct	Title	Multi-centre/ single centre	Sample Size	Field/ Topic	Ethnic/ Minority populations	Number of patients/ community representatives engaged in research	Methods of Engagement†			
								Development of research question	*Selecting outcome*	*Other activities*	*Dissemination and Implementation of results*
										London centre were invited to an annual patient and public involvement event at the Royal Brompton Hospital (once their direct involvement in the trial was over) to provide feedback on their	

ADDITIONAL FILE 2

Table 2. Full study characteristics of included studies.

† Denotes where excerpts are taken verbatim from study.

**Study**	**Country of conduct**	**Title**	**Multi-centre/ single centre**	**Sample Size**	**Field/ Topic**	**Ethnic/ Minority populations**	**Number of patients/ community representatives engaged in research**	**Methods of Engagement†**			
								Development of research question	*Selecting outcome*	*Other activities*	*Dissemination and Implementation of results*
										experiences. This feedback was collated and has been used to improve the study facilities at the site, as well as trial logistics, for example increased time for travel between sites.	

ADDITIONAL FILE 2

Table 2. Full study characteristics of included studies.

† Denotes where excerpts are taken verbatim from study.

**Study**	**Country of conduct**	**Title**	**Multi-centre/ single centre**	**Sample Size**	**Field/ Topic**	**Ethnic/ Minority populations**	**Number of patients/ community representatives engaged in research**	**Methods of Engagement†**			
								Development of research question	*Selecting outcome*	*Other activities*	*Dissemination and Implementation of results*
Gaucher, S., 2016, [43]	France	Assessment of a Standardized Pre-Operative Telephone Checklist Designed to Avoid Late Cancellation of Ambulatory Surgery: The AMBUPROG Multicenter Randomize	Multiple sites	4074	Ambulatory Surgery	Not reported	24	Not reported	Not reported	During the development of the intervention a checklist was created. "Given the reported effectiveness of checklists in reducing surgical complications and mortality [12], we developed a	Not reported

ADDITIONAL FILE 2

Table 2. Full study characteristics of included studies.

† Denotes where excerpts are taken verbatim from study.

Study	Country of conduct	Title	Multi-centre/ single centre	Sample Size	Field/ Topic	Ethnic/ Minority populations	Number of patients/ community representatives engaged in research	Methods of Engagement†			
								Development of research question	*Selecting outcome*	*Other activities*	*Dissemination and Implementation of results*
		d Controlled Trial								standardized pre-operative checklist that could be delivered to each patient via an automated phone system."	
Fraser, R., 2015 [44]	USA	PACES in epilepsy: Results of a self-management randomized	Single site	92	Epilepsy	Black, Latino, Asian, Other (unspecified)	50 surveys (165 returned)+ 2 focus groups of 10 patients + 22 adults	Not reported	Not reported	The survey was then refined by two focus groups of 10 adults with	Not reported

ADDITIONAL FILE 2

Table 2. Full study characteristics of included studies.

† Denotes where excerpts are taken verbatim from study.

**Study**	**Country of conduct**	**Title**	**Multi-centre/ single centre**	**Sample Size**	**Field/ Topic**	**Ethnic/ Minority populations**	**Number of patients/ community representatives engaged in research**	**Methods of Engagement†**			
								Development of research question	*Selecting outcome*	*Other activities*	*Dissemination and Implementation of results*
		controlled trial					with epilepsy from a local affiliate group			epilepsy, each at the University of Washington -Harborview Medical Center. The survey was then mailed to 250 adults with epilepsy who were being treated at either the UW-Harborview Medical	

ADDITIONAL FILE 2

Table 2. Full study characteristics of included studies.

† Denotes where excerpts are taken verbatim from study.

**Study**	**Country of conduct**	**Title**	**Multi-centre/ single centre**	**Sample Size**	**Field/ Topic**	**Ethnic/ Minority populations**	**Number of patients/ community representatives engaged in research**	**Methods of Engagement†**			
								Development of research question	*Selecting outcome*	*Other activities*	*Dissemination and Implementation of results*
										Center or the Swedish Medical Center, and a group of 22 adults with epilepsy in local affiliate support groups. The survey participants in the mailing were screened for marked	

ADDITIONAL FILE 2

Table 2. Full study characteristics of included studies.

† Denotes where excerpts are taken verbatim from study.

**Study**	**Country of conduct**	**Title**	**Multi-centre/ single centre**	**Sample Size**	**Field/ Topic**	**Ethnic/ Minority populations**	**Number of patients/ community representatives engaged in research**	**Methods of Engagement†**			
								Development of research question	*Selecting outcome*	*Other activities*	*Dissemination and Implementation of results*
										cognitive or emotional impairment by each center's epileptologists. The Dillman approach was used in order to optimize participant response.17 A total of 165 returns were received (61% response	

ADDITIONAL FILE 2

Table 2. Full study characteristics of included studies.

† Denotes where excerpts are taken verbatim from study.

Study	Country of conduct	Title	Multi-centre/ single centre	Sample Size	Field/ Topic	Ethnic/ Minority populations	Number of patients/ community representatives engaged in research	Methods of Engagement†			
								Development of research question	*Selecting outcome*	*Other activities*	*Dissemination and Implementation of results*
										rate). There was very high response reliability (internal consistency) across all domains in the survey. A comprehensive description of the survey findings is available in Fraser et al., with	

ADDITIONAL FILE 2

Table 2. Full study characteristics of included studies.

† Denotes where excerpts are taken verbatim from study.

Study	Country of conduct	Title	Multi-centre/ single centre	Sample Size	Field/ Topic	Ethnic/ Minority populations	Number of patients/ community representatives engaged in research	Methods of Engagement†			
								Development of research question	*Selecting outcome*	*Other activities*	*Dissemination and Implementation of results*
										implications for the development of the PACES program"	
Gimeno-Santos, E., 2015 [45]	Spain	The PROactive instruments to measure physical activity in patients with chronic obstructive pulmonary disease	Multiple sites	236	COPD	Not reported	Not reported	Not reported	Not reported	Within the framework of the European Union Innovative Medicines Initiative PROactive project (www. proactivecopd.com) our group first	Not reported

ADDITIONAL FILE 2

Table 2. Full study characteristics of included studies.

† Denotes where excerpts are taken verbatim from study.

**Study**	**Country of conduct**	**Title**	**Multi-centre/ single centre**	**Sample Size**	**Field/ Topic**	**Ethnic/ Minority populations**	**Number of patients/ community representatives engaged in research**	**Methods of Engagement†**			
								Development of research question	*Selecting outcome*	*Other activities*	*Dissemination and Implementation of results*
										conducted qualitative research and drafted a conceptual framework to set out the concept of physical activity from patients' experience and to provide the necessary basis to generate an item pool for two	

ADDITIONAL FILE 2

Table 2. Full study characteristics of included studies.

† Denotes where excerpts are taken verbatim from study.

Study	Country of conduct	Title	Multi-centre/ single centre	Sample Size	Field/ Topic	Ethnic/ Minority populations	Number of patients/ community representatives engaged in research	Methods of Engagement†			
								Development of research question	*Selecting outcome*	*Other activities*	*Dissemination and Implementation of results*
										PROs, one with a daily recall period and one with a 7-day recall period (daily and clinical visit versions, respectively) to measure the experience of physical activity in patients with COPD. The study	

ADDITIONAL FILE 2

Table 2. Full study characteristics of included studies.

† Denotes where excerpts are taken verbatim from study.

**Study**	**Country of conduct**	**Title**	**Multi-centre/ single centre**	**Sample Size**	**Field/ Topic**	**Ethnic/ Minority populations**	**Number of patients/ community representatives engaged in research**	**Methods of Engagement†**			
								Development of research question	*Selecting outcome*	*Other activities*	*Dissemination and Implementation of results*
										was also approved by patient advisory boards and the advisory board provided advice to guide the whole process for the item reduction and validation of the tools. Dobbels F, de Jong C, Drost E, et	

ADDITIONAL FILE 2

Table 2. Full study characteristics of included studies.

† Denotes where excerpts are taken verbatim from study.

Study	Country of conduct	Title	Multi-centre/ single centre	Sample Size	Field/ Topic	Ethnic/ Minority populations	Number of patients/ community representatives engaged in research	Methods of Engagement†			
								Development of research question	*Selecting outcome*	*Other activities*	*Dissemination and Implementation of results*
										al. The PROactive innovative conceptual framework on physical activity. Eur Respir J 2014; 44: 1223–1233.	
Kattelmann, K., 2014 [23]	USA	The Effects of Young Adults Eating and Active for Health (YEAH): A Theory-Based Web-	Single site	1639	Nutrition and exercise	African-American, Asian, Native Hawaiian/Pacific Islander, American Indian,	Not reported	Not reported	Not reported	The intervention was developed us- ing the CBPR process of PRECEDE-PROCEED and was	Not reported

ADDITIONAL FILE 2

Table 2. Full study characteristics of included studies.

† Denotes where excerpts are taken verbatim from study.

Study	Country of conduct	Title	Multi-centre/ single centre	Sample Size	Field/ Topic	Ethnic/ Minority populations	Number of patients/ community representatives engaged in research	Methods of Engagement†			
								Development of research question	*Selecting outcome*	*Other activities*	*Dissemination and Implementation of results*
		Delivered Intervention				Hispanic, and other				delivered via the Web and e-mail. Greene GW, White AA, Hoerr SL, et al. Impact of an online healthful eating and physical activity program for college students. Am J Health Promot.	

ADDITIONAL FILE 2

Table 2. Full study characteristics of included studies.

† Denotes where excerpts are taken verbatim from study.

**Study**	**Country of conduct**	**Title**	**Multi-centre/ single centre**	**Sample Size**	**Field/ Topic**	**Ethnic/ Minority populations**	**Number of patients/ community representatives engaged in research**	**Methods of Engagement†**			
								Development of research question	*Selecting outcome*	*Other activities*	*Dissemination and Implementation of results*
										2012;27:E47-E58.	
